# The cross-linguistic comparison of perceptual strength norms for Korean, English and L2 English

**DOI:** 10.3389/fpsyg.2023.1188909

**Published:** 2023-07-19

**Authors:** Jonghyun Lee, Jeong-Ah Shin

**Affiliations:** ^1^Department of English Language and Literature, College of Humanities, Seoul National University, Seoul, Republic of Korea; ^2^Department of English Language and Literature, College of Humanities, Dongguk University, Seoul, Republic of Korea

**Keywords:** perceptual strength, conceptualization, embodiment cognition, grounded cognition, cross-linguistic, second language processing, interoception, abstract concept

## Abstract

This study aimed to establish perceptual strength norms for 1,000 words in the languages of Korean, English, and L2 English, in order to investigate the similarity and difference across languages as well as the influence of the environment on semantic processing. The perceptual strength norms, which are a collection of word profiles that summarize how a word is experienced through different sensory modalities including the five common senses and interoception, provide a valuable tool for testing embodiment cognition theory. The results of this study demonstrated that language users had parallel sensory experiences with concepts, and that L2 learners were also able to associate their sensory experiences with linguistic concepts. Additionally, the results highlighted the importance of incorporating interoception as a sensory modality in the development of perceptual strength norms, as it had a negative correlation with both vision and concreteness. This study was the first to establish norms for Korean and L2 English and directly compare languages using the identical and translation-equivalent word list.

## 1. Introduction

The embodied or grounded cognition theory provides a contrast perspective from the traditional approaches to semantic processing. In the conventional framework, semantic processing, the comprehension and interpretation of language, is typically viewed as an abstract computational process (Quillian, 1967; Fodor, 1983). This process relies primarily on the symbolic representations that are stored in memory, isolated from our sensory and motor experiences in an abstract form (Murphy, 2004; Barsalou, 2008). Investigations within this perspective focused on the organization of concepts in memory, either by scrutinizing the relationships between them (Quillian, 1967; Collins and Loftus, 1975) or assessing their shared and unique features (Farah and McClelland, 1991; McRae et al., 1997).

However, during recent decades, a shift has occurred in theoretical orientation towards an embodied approach, which has been derived from the Dual Coding Theory (DCT; Paivio, 1991, 2013). DCT is a cognitive theory that suggests semantic information is processed by two separate, yet interconnected systems: a verbal and a nonverbal system (Dove, 2011). According to this view, semantic processing is not solely reliant on the verbal system, but also necessitates the nonverbal system, implying that semantic representations maintain certain nonverbal aspects of the external sensory experiences from which they originate (Sadoski, 2015). In a similar vein, the embodied perspective posits that semantic representations are intimately connected to sensory, motor, and introspective experiences and are formed by the reactivation or simulation of these multi-modal experiences in the brain (Barsalou, 2008, 2010; Dijkstra and Post, 2015). In this context, a growing body of research has observed that semantic representations depend on multi-modal experiences and are associated with activations in multiple brain regions, including the sensory and motor cortex (Glenberg and Kaschak, 2002; Chambers et al., 2004; Hauk et al., 2004; Hauk and Pulvermüller, 2004; Pulvermüller et al., 2005, 2009; Tettamanti et al., 2005; Glenberg et al., 2008; Bergen et al., 2010; Vukovic and Shtyrov, 2014; Klepp et al., 2015; Cervetto et al., 2021). Despite the increasing evidence favoring embodied cognition theory, a more rigorous examination of multimodal experiences is necessary to further solidify its foundations. This investigation calls for the employment of adequate stimuli, such as those created using perceptual strength norms, thereby facilitating more nuanced empirical studies (Lynott et al., 2020; Miceli et al., 2021). The perceptual strength norm is a collection of word profiles that summarize through which senses a word is experienced and how strongly it is perceived through those senses. The evaluation process for perceptual strength norm, though seemingly similar to concreteness rating in that both evaluate a word employing a Likert scale, distinctively categorizes multiple sensory perceptions rather than aggregating them into one concreteness measure.

The utilization of the perceptual strength norm, which entails the separate evaluation of sensory modalities, has several benefits in the process of norming the sensory foundation of concepts. One of the most notable advantages is that it aligns with neuroanatomical basis of sensory perception. The primary sensory cortex consists of separate cortical regions corresponding to each sensory modality (Marieb and Hoehn, 2007; Glasser et al., 2016), and these are activated by the associated concepts or words. For instance, the primary auditory cortex, located on the top boundary of the temporal lobe, and the primary visual cortex, situated at the occipital lobe’s extreme posterior tip, become more activated for words or concepts with strong auditory (Kiefer et al., 2008, 2012; Trumpp et al., 2013, 2014) or visual (Simmons et al., 2007; Harpaintner et al., 2020) perceptual strengths, respectively (Gustatory: Barros-Loscertales et al., 2012; Olfactory: González et al., 2006). Another advantage is the ability to isolate the effects specific to certain sensory modalities, which enables researchers to conduct more precise and granular investigations on individual sensory modalities (Lynott et al., 2020). For example, Connell and Lynott (2014) showed that performance in lexical decision tasks, which rely heavily on visual attention, was strongly correlated with visual perceptual strength, whereas both auditory and visual strength were reliable predictors for performance in reading aloud tasks which requires auditory attention. Additionally, modality-specific measures are reliable predictors of performance on cognitive tasks such as lexical decision (Connell and Lynott, 2012, 2014; Juhasz and Yap, 2013), word naming (Connell and Lynott, 2012, 2014), and property verification (Lynott and Connell, 2009; Collins et al., 2011; Hald et al., 2011; Louwerse and Connell, 2011; Lynott et al., 2020). For example, Connell and Lynott (2012) found that modality-specific experience was a more reliable predictor of performance than concreteness or imageability. It might be because rating sensory experience collectively, rather than focusing on individual modalities, leads to a loss of information and weaker semantic facilitation (Connell and Lynott, 2016).

Taking advantage of these benefits, perceptual strength norms have been developed in a variety of languages, including English (Lynott et al., 2020), Russian (Miklashevsky, 2018), Dutch (Speed and Majid, 2017; Speed and Brybaert, 2022), Italian (Vergallito et al., 2020), French (Chedid et al., 2019; Miceli et al., 2021), and Mandarin (Chen et al., 2019). However, to date, no such investigation has been conducted in Korean or for second language (L2) learners. The primary objective of the present study thus is to establish, for the first time, perceptual strength norms for Korean and L2 learners.

The establishment of perceptual strength norms for novel languages and learners not only serves as appropriate stimuli for research on the users of that language, but also facilitates the examination of the interactions between concepts and environments through cross-linguistic comparisons. According to the embodiment cognition theory, the formation of embodiment is greatly influenced by the repetitive association between concept and environment (Pulvermüller, 2005). Therefore, as the embodiment of a concept may vary depending on the learning environment, the corresponding perceptual experience may also differ. Korean, the focus of the present study, is an under-researched Asian language, which has a distinct cultural background compared to the European languages that have been the subject of numerous previous studies. The same concept might be perceived differently by speakers of Korean and English, leading to variations in the perceptual strengths. Previous research has shown that the distribution or predictability of perceptual strengths varies across languages (Vergallito et al., 2020; Miceli et al., 2021). However, those findings were based on indirect comparisons between perceptual strength norms generated by individual studies. It must be noted that even within the same language, perceptual strengths can vary depending on the method or word set (Speed and Majid, 2017; Speed and Brybaert, 2022), and thus, such indirect comparisons may not accurately reflect variations in perceptual strengths and interaction between concept and environment. In contrast to prior investigations, the present study conducts a direct comparison of three language groups (Korean, English, and L2 English) by evaluating the perceptual strengths of a common set of 1,000 written words across all groups.

This study incorporates the perceptual strengths of L2 learners in cross-linguistic comparisons, because this may have interesting implications in terms of the influence of the environment on the sensory experience of a concept. Since L2 English participants in this experiment learn English as a foreign language in Korea, the environment surrounding their linguistic concepts is more congruent with that of L1 Korean participants. As a result, when they assess L2 English perceptual strengths, their sensory experience may be more similar to that of Korean speakers, even though they are processing English on the surface, leading to their perceptual strengths being more aligned with those of Korean speakers. It should be noted, however, that the factors influencing L2 processing are multifaceted, which can result in a more intricate pattern in the actual norms. Bilingual lexicon models for L2 lexical processing (Dong et al., 2005; Pavlenko, 2009) posit that the conceptual representation of L2 lexicon is connected not only to an L1 concept (native language) but also to an L2 concept (target language). Depending on the lexical item or the learners’ proficiency, the associated concepts may be more strongly linked to L2 than the learners’ native language, regardless of the environment surrounding them. From this point of view, examining the patterns of L2 English learners’ perceptual strengths, whether they are close to that of Korean or English, can provide new insights into the exploration of the effect of the environment on semantic processing.

Most of previous studies establishing perceptual strength norms employed five common senses: visual, auditory, tactile, olfactory and gustatory sense. However, the present study adds another sense—interoception. It is one of most important senses for human beings, but it is often overlooked in the sensory-motor classification. Interoception pertains to perception to the internal state of the body, which includes muscular and visceral sensations, vasomotor activity, hunger, thirst, breathlessness, pain, pleasure, temperature, itch, and sensual touch (Craig, 2003; Craig, 2011). Interoception has been suggested as an important sensory modality for studying the embodiment of abstract concepts (Dove et al., 2020), because it plays an important role in the processing of emotions, which are related with various abstract concepts (Critchley and Harrison, 2013; Connell et al., 2018). Previous research on perceptual strength norms (Lynott et al., 2020; Miceli et al., 2021) has incorporated interoception as a crucial sensory modality. These studies demonstrated that participants could effectively engage their interoceptive perception or were able to rate words based on the interoceptive experiences. Moreover, Lynott et al. (2020) identified a negative correlation between interoception and vision. Considering the established positive correlation between vision and concreteness (Connell and Lynott, 2012; Vergallito et al., 2020), intriguing questions emerge regarding the potential link between interoception and concreteness. Even though an association between interoception and abstractness has been suggested, the correlation between interoception and concreteness remains largely unexplored in existing literature. Our study thus seeks to address this gap, aiming not only to corroborate the previous findings of effective engagement of interoception by participants but also to explore the largely uninvestigated relationship between concreteness and interoception. This endeavor is anticipated to contribute to a more holistic comprehension of how sensory modalities might interact with the concreteness of language.

Given the aforementioned context, the present study aims to: (i) Develop perceptual strength norms of 1,000 words for the research on Korean and L2 English learners. (ii) Compare the perceptual strengths of L1 English, L1 Korean, and L2 English speakers for the identical words, in order to investigate the effect of cultural environment on semantic processing of concepts. (iii) Evaluate the effectiveness of interoception and examine its correlation with concreteness.

## 2. Materials and methods

### 2.1. Materials

A total of 1,000 English words (Supplementary material for the word list) were selected from about 40,000 words used in The Lancaster Sensorimotor Norms (Lynott et al., 2020) considering familiarity, word frequency, max perceptual strength, exclusivity, and translatability. On the basis of these criteria, words were chosen that were easily understood by L2 English speakers (frequency and familiarity), were straightforward to translate into Korean (translatability), and may serve as the experimental material for future neurolinguistic studies (max perceptual strength, exclusivity). Word frequency was measured with the Python package WordFreq (Speer et al., 2018). Only the words with frequency of greater than 3.5 on the Zipf scale were included. Max strength refers to the score of the sense with the highest one among the six senses (visual, auditory, haptic, gustatory, olfactory, and interoceptive). Exclusivity is the extent to which a concept is experienced through a single sense, calculated by dividing the sum of all sensory scores by the max strength. Only words with above-average max strength and exclusivity within each sense were selected because words with similar scores in several senses (low exclusivity) or words with low perceptual strength (low max strength) may not elicit distinct neural activities, thus less suitable for use in future research. Also, the words that cannot be easily translated into Korean were eliminated, such as polysemy, prepositions, pronouns, and onomatopoeia.

A Korean item set consisted of Korean translation equivalents of the English set. Translations into Korean were created based on the experience of language users rather than dictionary definitions. This was to narrow the gap between the meaning of a word commonly recognized by L2 users and its dictionary definition, as well as to meet the purpose of this experiment measuring the sensory experience of a concept. For the translation process, 11 participants were recruited as translators. Since they were also L2 learners who had similar levels of English proficiency, academic background, and age as the participants in the main experiment, they were expected to have similar sensory experiences for words. Each of them translated 1,000 English words into Korean. They were instructed to translate each English word into the Korean word that sprang to mind, that is, to use their experience of words. If a word has multiple meanings with several possible translation candidates, they were forced to choose the first one that they recalled. Translations into loanwords were permitted, as they were commonly accepted as part of the Korean vocabulary. Moreover, they could serve as a testbed for examining differences between languages. Nevertheless, when their first translation was a loanword, they were asked to come up with another possible translation that was not a loanword and to select the one that was more common in the context of everyday life between them. This was to rule out simple transliteration of English words or the cases that the translated words were more likely to be recognized as a foreign language rather than Korean. For translation they were not allowed to look up a dictionary or other reference materials. When they did not know the meaning of a word, they were instructed to skip it over. After collecting the translation candidates from the translators, the translation agreed upon by the most translators was selected as the representative translation. If more than one translation tied the agreement voting, the one closer to the first definition in the English-Korean dictionary was chosen by the experimenter’s judgment.

For the concreteness analysis, we employed the widely recognized concreteness ratings from Brysbaert et al. (2014), developed for English words. For the Korean words, concreteness values were assigned by referencing their English translations. This approach might not entirely capture the concreteness nuances in Korean due to cultural variances, given the findings of the cross-linguistic correlations in concreteness ratings, for instance, between English and Japanese (Allen and Conklin, 2014) and English and Russian (Marian and Kaushanskaya, 2007), this approach might serve as a tool to explore general trend, particularly those concerning the correlation between sensory modalities and concreteness. Nevertheless, the findings should be interpreted with caution.

### 2.2. Participants

For the L2 English dataset, a total of 55 unique participants (male: 21, female: 34; age: mean = 26.78, SD = 3.63, range = 21 to 38) were recruited for the experiment. They completed on average 3.51 lists each. They were Korean learners of L2 English, who were born in Korea. Their English proficiency level was advanced according to the standardized test (TEPS: Test of English Proficiency developed by Seoul National University). Each word list was rated by 19.3 participants on average, and all words were rated by at least 18 participants. For the L1 Korean dataset, 48 participants (male: 19, female: 29; age: 26.51, SD = 3.50, range = 19 to 36) completed the survey, and each evaluated 4.40 lists on average. Their English proficiency was also advanced level. Each word list was rated by an average of 21 participants, each rated by at least 19 participants. All they were recruited from the university website. For L1 English survey, 173 unique participants (male: 93, female: 80; age: mean = 40.38, SD = 11.47, range = 21 to 78) were recruited from Amazon Mechanical Turk.^1^ Participation was restricted to only those who speak English as their first language and reside in an English-speaking country. They performed an average of 1.04 list. Each word was rated by an average of 19.1 participants and by at least 18. While it was an option to use the results from Lynott et al. (2020) as a reference for L1 English perceptual strength norms, this study opted to collect new data from native English speakers for this study. This was to avoid potential influences introduced due to the different word sets and evaluation items in Lynott et al.’s (2020) study, which included around 40,000 words and five action effectors along with six perceptual modalities. This approach might help ensure the integrity of our evaluations and maintain the validity of direct comparisons. All the participants were provided with a monetary reward for their participation in the study. The participant count described above pertains to the final sample and does not include those who were subsequently excluded based on our criteria (details in “Analysis”).

### 2.3. Procedure

The surveys were created using PCIbex (Zehr and Schwarz, 2018) and conducted online. Since it was difficult for one participant to evaluate all the items, 1,000 words, at once, they were randomly divided into 10 sets of 100 words. Participants were free to choose which sets they would perform and allowed to participate in more than one word list, but not in the same list twice. After selecting the set, participants read the outline of the experiment and started it if they agreed to participate in it. They learned the procedure through the instruction and five practice trials. After practice, they performed the main task, which took an average of 25 min for the completion. The tasks for Korean participants were presented in Korean to help participants understand accurately. All procedure was approved by and conducted in accordance with SNUIRB (the ethical committee of Seoul National University).

During the task, participants were asked to evaluate the sensory experience and word familiarity on English or Korean words. Each word first appeared on the screen, followed by rating scales located below the word. Above scales were the labels of senses evaluated. Participants rated how strongly they used each sense in experiencing a particular word. The senses evaluated were visual, auditory, haptic, gustatory, olfactory, and interoceptive. The definition of interoceptive sense was given in the instruction and during the task. Through the instruction, it was explained that there was no right or wrong answer, so evaluation should be based on one’s own intuition. A 6-point Likert scale was used ranging from 0 (not experiencing the sense at all) to 5 (experiencing the sense strongly). Subsequent to the perceptual strength evaluation, participants assessed word familiarity. This involved determining their level of familiarity with the word and its meaning, using a 4-point Likert scale (not at all 0—very well 3). They were asked whether they are familiar with the word or whether they know what it means. This measure aimed to identify whether the L2 participants were acquainted with the respective words.

The five practice trials presented before the main task were not only a means to assist in understanding of the task, but also functioned as calibrator words. They played a role in allowing participants to employ various sensory dimensions over the entire range through words with relatively clear sensory indices. The same five calibrators with those in Lynott et al. (2020) were used, which were *account* (Low strength across all modalities), *breath* (medium strength across multiple modalities), *echo* (high strength in a single modality), *hungry* (uneven strength across modalities) and *liquid* (high strength across multiple modalities).

### 2.4. Analysis

For the analysis, first, participants who consistently assigned the same score to all items were deemed to have not adequately performed the task and were excluded from the analysis. This led to the removal of 13 participants from the L1 English group. Additionally, any participant whose overall average word familiarity was below 2 was also planned to be excluded, although none of the participants met this criterion. Then, words with low average familiarity (below 2) and individual rated items with low familiarity (below 2) were excluded from the analysis (L1 Korean: 11 words, 3.41% of the data, L2 English: 29 words, 3.85%, L1 English: 0 word, 1.12%). Trimmed data were averaged by each word and sensory modality. The analysis consisted of two stages. First, summary statistics of perceptual strengths of words were processed in various ways to analyze similarities and differences between modalities and languages. In the results section, the *interrater reliability, perceptual strength, correlation and distance, dominant modality, exclusivity, relationship between modalities* and *relationship between modalities and concreteness* were presented and briefly discussed. To explore the mean differences between languages for each modality, a mixed-effects linear regression model was employed, using lme4 (Bates et al., 2015) packages in R. The model accounted for fixed effects of age and group (L1 English, L2 English, L1 Korean). Age was included in the model to control for potential age-related influences. Individual participants, words and word list were treated as random effects, addressing variability across these factors. As a first step in the analysis, a type III ANOVA was run on the fitted mixed-effects model to test for differences in sensory perception strength across the language groups. If significant (*p* < 0.05), this was followed by pairwise comparisons using Tukey’s method, controlling the familywise error rate among multiple tests.

Second, selected individual words were analyzed to highlight the differences between languages that were not revealed by quantitative analysis alone. Several intriguing cases with implications for conceptual differences or environmental influences were selected through visual inspection on the radar charts of perceptual strengths, considering various factors such as polysemy, usage, translation agreement, and loanwords. In particular, words with high translation concordance and loanwords were the primary focus of analysis, as they tended to mitigate the challenges in comparison presented by the lack of complete one-to-one correspondence between Korean and English. For example, *eagle* was translated identically in Korean (‘toksuli’) by all translators. Even if the usage and experience of these words might vary between languages, they were at least matching in form. The same held true for loanwords, which even exhibited a high degree of phonetic similarity and shared common etymological origins across languages. These findings are described in *individual cases* and *individual cases for loanwords* section.

## 3. Results

### 3.1. Interrater reliability

Interrater reliability for perceptual strengths was calculated using Cronbach’s alpha, which is a measure of internal consistency. It was calculated per item list, using a python statistical package, Pingouin (Vallat, 2018), and then averaged within each language. All language groups showed excellent interrater reliability for all modalities (*α* > 0.9; Table 1A). Each Cronbach’s alpha level in this study was comparable to that of Lynott et al. (2020). This suggested that perceptual strengths evaluated by participants were not arbitrary but internally consistent, reflecting what they evaluated.

**Table 1:** **(A)** Mean Cronbach’s alpha for each modality within each language group. Larger than 0.9 generally means excellent internal consistency. **(B)** Mean perceptual strength ratings (0–5) and standard deviations (SD). **(C)** The correlation scores across languages.

		Auditory	Gustatory	Haptic	Interoceptive	Olfactory	Visual
**(a) Interrater reliability (Cronbach’s alpha)**							
Korean	L1	0.98	0.95	0.96	0.98	0.95	0.98
English	L1	0.98	0.92	0.96	0.97	0.93	0.96
	L2	0.98	0.98	0.98	0.98	0.98	0.98

**(b) Perceptual strength ratings**							
Korean	L1	1.74 (1.77)	0.29 (0.90)	1.26 (1.61)	0.94 (1.49)	0.37 (0.92)	3.91 (1.33)
English	L1	2.06 (2.00)	0.28 (0.95)	1.57 (1.88)	1.06 (1.66)	0.33 (0.95)	3.93 (1.59)
	L2	1.69 (1.70)	0.33 (0.88)	1.29 (1.54)	1.16 (1.51)	0.40 (0.89)	3.70 (1.35)

**(c) Correlations between languages**						
L1E vs. L2E	0.81^***^	0.91^***^	0.82^***^	0.83^***^	0.83^***^	0.81^***^
L1E vs. L1K	0.74^***^	0.87^***^	0.73^***^	0.80^***^	0.80^***^	0.75^***^
L2E vs. L1K	0.88^***^	0.90^***^	0.85^***^	0.84^***^	0.84^***^	0.84^***^

Larger than 0.7 is generally considered strong. All coefficients are significant (*p* < 0.001).

### 3.2. Perceptual strengths

**Figure 1:**
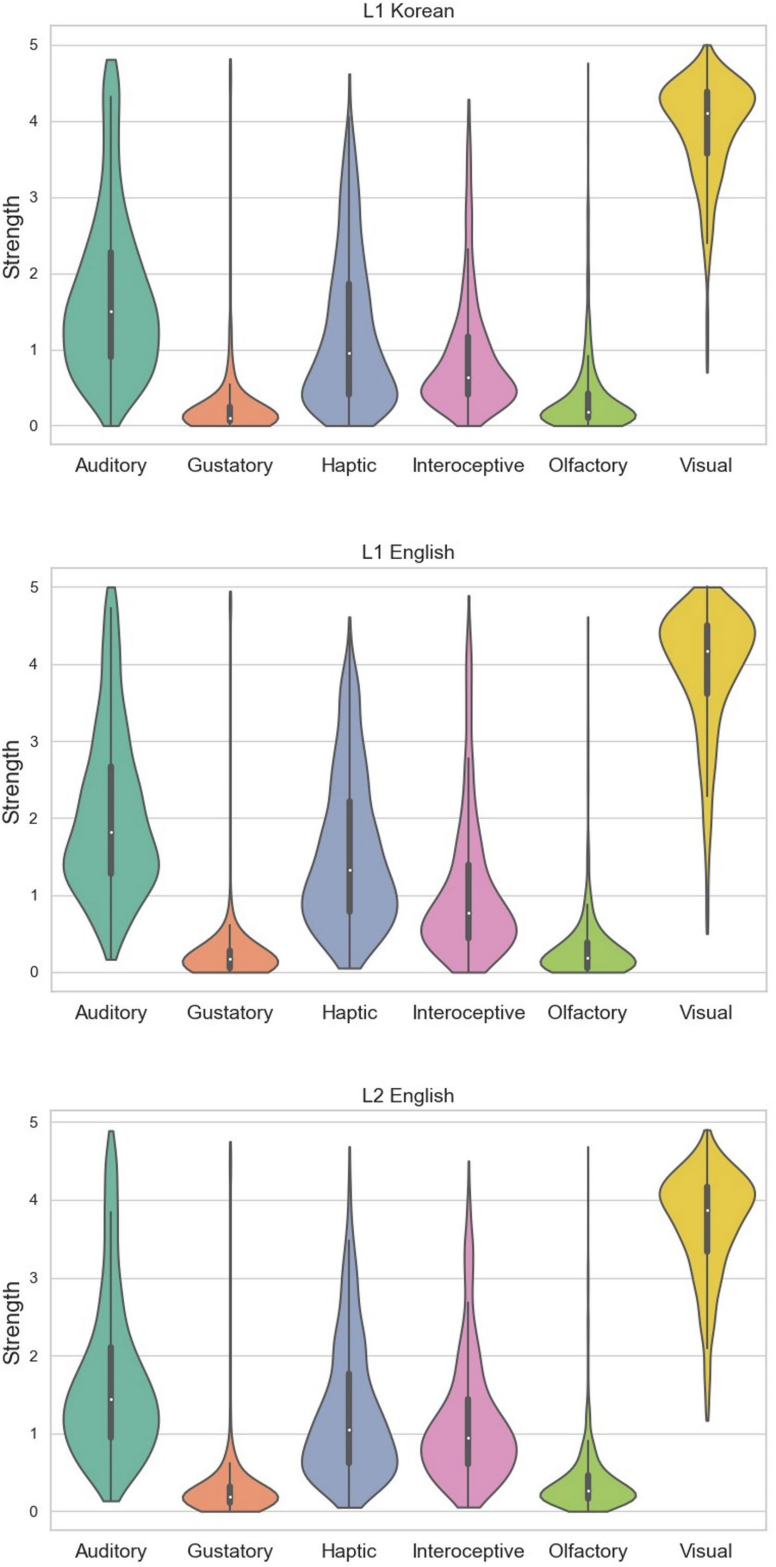
Violin plots showing the distribution of perceptual strength ratings for each sensory modality. Black lines and boxes inside indicating boxplots. The three groups have similarity in shape and distributions. Top: L1 Korean. Middle: L1 English. Bottom: L2 English.

Summary statistics (mean and standard deviations) per modality are shown in Table 1B and Figure 1. The general patterns and distributions of perceptual strengths in L1 Korean, L1 English, and L2 English exhibited similarities. In all languages, vision was rated the highest, while olfactory and gustatory were low. This distribution was almost similar to that from other perceptual strengths norm in previous studies such as Lynott et al. (2020).

In comparing perceptual strengths across languages, there were no significant differences between languages in the visual, olfactory, gustatory, and interoceptive modalities (*p* > 0.05). However, for the haptic modality, a significant effect was found for the language group (*F*(2, 47.76) = 3.96, *p* < 0.05). Pairwise comparisons revealed significant differences between L1 English and L1 Korean (estimate = 0.3264, SE = 0.1247, *t* = 2.62, *p* < 0.05), and between L1 English and L2 English (estimate = 0.3367, SE = 0.1196, *t* = 2.81, *p* < 0.05). However, there was no significant difference between L1 Korean and L2 English (*p* > 0.05). In the auditory modality, the language group demonstrated a significant effect (*F*(2, 43.309) = 3.66, *p* < 0.05). The *post-hoc* analysis again showed a significant difference between L1 English and L1 Korean (estimate = 0.4316, SE = 0.1614, *t* = 2.68, *p* > 0.05), and between L1 English and L2 English (estimate = 0.4274, SE = 0.1678, *t* = 2.55, *p* < 0.05). No significant difference was observed between L1 Korean and L2 English (*p* > 0.05).The correlations between languages for each modality were measured to see whether language groups had similar distribution. The correlations were computed by using as variables the mean perceptual strengths for each modality per word (average of perceptual strengths rated by each participant). As a result, there was overall a strong correlation between languages in all modalities (Table 1C). In general, the correlation coefficients between L1 English and L1 Korean were lower than L2 English and L1 Korean or L1 English and L2 English.

In addition, Euclidean distances between languages were calculated to measure similarities between language groups. The Euclidean distance is the way to determine the distance between two points, often used as a measure of similarity. The distances between languages were computed in a pairwise manner, resulting in a vector of the distances for the mean perceptual strengths of each word between two language groups. For instance, the mean perceptual strengths of the word, addiction, were “[auditory: 1.5, Gustatory: 1.4, Haptic: 1.11, Interoceptive: 3.22, Olfactory: 0.89, Visual: 3]” in L1 English, and “[auditory: 1.26, Gustatory: 0.74, Haptic: 1.89, Interoceptive: 2.95, Olfactory: 1.58, Visual: 2.58]” in L2 English. The distance between these two points was calculated as 0.93. These word distances could be obtained for all words in two language groups, which made up a vector of the word distances. The mean of the word distances between languages were relatively small for all comparisons (L1 English vs. L2 English: 1.22, L1 English vs. L1 Korean: 1.38, L1 Korean vs. L2 English: 0.97), compared to the distances between each language and randomly created perceptual strengths, which were about 5 on average. This indicated that there are similarities between languages. L1 Korean and L1 English showed the lowest similarity among them, which was consistent with the findings that they had the weaker correlations. On the other hand, L1 Korean and L2 English had the highest similarity.

In summary, the distributions of perceptual strengths in three groups were similar to those in previous studies—higher visual rate and lower olfactory and gustatory. Also, although there were some differences in each rating, considering the correlation and Euclidean distance, the overall distribution across all language groups seemed to be similar.

### 3.3. Dominant modalities

The dominant modality for a word is the one with the highest rating among six sensory modalities (Table 2). When two modalities tied the highest rating, both were treated as dominant modalities. For example, as finger was rated as 4.10 both in haptic and visual sense by L2 English participants, it was labeled as Haptic-Visual dominant words and counted, respectively, as a haptic-dominant word and a visual-dominant word.

**Table 2:** Numbers and percentage of dominant modalities within L1 Korean, L1 English and L2 English.

		Auditory	Gustatory	Haptic	Interoceptive	Olfactory	Visual
Korean	**L1**	73 (7.97%)	15 (1.64%)	6 (0.66%)	32 (3.49%)	1 (0.11%)	862 (87.16%)
English	**L1**	98 (9.73%)	18 (1.79%)	11 (1.09%)	60 (5.96%)	3 (0.30%)	817 (81.13%)
	**L2**	81 (8.28%)	17 (1.74%)	10 (1.02%)	59 (6.03%)	2 (0.20%)	809 (82.72%)

The distributions of dominant modality were also similar across languages. For all language groups, vision was the most dominant sensory dimension, while the proportion of olfactory, haptic and gustatory-dominance was relatively low. L1 Korean appeared to be distinguished from the others in that it was more visually dominant and less dominant in touch and interoception. Words rated as haptic and interoceptive words in L1 English and L2 English were mostly evaluated as visual words in L1. However, those rated vision-dominant in L1 Korean were not experienced in a completely different way, since in most cases their second dominant modality was haptic or interoception. In short, the words with haptic or interoceptive dominance in L1 English and L2 English were often visually dominant in L1 Korean, but their profile of perceptual strengths was not completely different from other language groups.

### 3.4. Exclusivity

Exclusivity scores per word are calculated by dividing the max perceptual strength with the sum of all strengths. It ranges from 0% (experienced equally in all senses) to 100% (solely through a single sense). A mean exclusivity score for L1 English was 46.37%, for L2 English 46.71% and for L1 Korean 49.84% (Table 3). According to the average exclusivity, L1 Korean was less multi-dimensional than the other two groups. The correlation coefficient of exclusivity between L1 English and L2 English was 0.67 (*p* < 0.001), between L1 Korean and L2 English was 0.75 (*p* < 0.001), between L1 English and L1 Korean was 0.59 (p < 0.001). The correlations were generally strong, and the lowest between L1 English and L1 Korean, similar to those in perceptual strengths.

**Table 3:** Exclusivity, 10 most exclusive words and 10 least exclusive words for L1 Korean, L1 English, and L2 English.

	Exclusivity	10 most exclusive words (dominant modalities)	10 least exclusive words (dominant modalities)
L1 Korean	49.84% (*sd = 11.08*)	shadow, moon, star, rainbow, graph, satellite, visual, cloud, cloud, lane (visual)	feel, hunger, pulse (interoceptive), drinking, pizza, cake, honey, butter (gustatory), happy, apple (visual)
L1 English	46.37% (*sd = 8.47*)	rainbow, white, online, moon, download, percentage, galaxy, email, mars, shadow (visual)	drinking, salad, lemon, apple, delicious, cake, honey, olive, juice, pizza (gustatory)
L2 English	46.71% (*sd = 9.10*)	brown, white, gray, moon, yellow, green, horizon, logo, black, diameter (visual)	feel (haptic), drinking (visual), healing (interoceptive), pizza, honey, apple, salad, addiction, cherry, rice (gustatory)

Listed first are the most exclusive or least exclusive words, but the rest may not be in order depending on dominant modalities.

In L1 English, *rainbow* was the least muti-modality word, with 82.46% exclusivity, while *drinking* was the least exclusive one, with exclusivity of 20.7%. Ten most exclusive words were all visual words, while ten least exclusive words were all gustatory words, which was often accompanied by vision, smell, or interoception. In L2 English, *brown* was the word with the highest exclusivity score (80.73%), and *feel* was the word with the lowest exclusivity score (20.78%). Ten highest words were also all visual words, whereas ten lowest words were mostly gustatory words but also included haptic, visual, and interoceptive words. In L1 Korean, *shadow* (88.69%) and *feel* (20.72%) was the highest and lowest exclusivity word. The most exclusive words were all visual dominant words, while the least ones were gustatory or interoceptive and visual words with high gustatory strengths. The most exclusive words were visually dominant in all three language groups. The ten words with high scores in each group mostly overlapped each other. The least exclusive words also had cross-linguistic overlap and were similar in that they were in most cases related to gustatory sense. However, L1 Korean and L2 English included several interoception and visual word such as *feel*, *healing*, and *happy*.

### 3.5. Relationship between modalities

The correlations between sensory modalities were calculated (Table 4). Similar to previous studies (Connell and Lynott, 2012; Vergallito et al., 2020), gustatory and olfactory strength were highly correlated, while visual and haptic strength had modest correlation. This was consistent with our intuition that one can experience both taste and smell in the intake of food, while touchable objects are often visible. On the other hand, auditory sensory showed a moderate negative correlation with vision and touch, while interoception was negatively correlated to vision. This was also compatible with our observation that audible objects cannot be seen or touched, nor can we see sensations inside the body.

**Table 4:** The correlation matrix between modalities.

		Auditory	Gustatory	Haptic	Interoceptive	Olfactory	Visual
Auditory	L1K		−0.06	−0.29^***^	0.12^***^	−0.02	−0.32^***^
	L1E		−0.17^***^	−0.41^***^	0.07	−0.19^***^	−0.36^***^
	L2E		−0.08	−0.32^***^	0.11^***^	−0.07	−0.31^***^
Gustatory	L1K	−0.06		0.17^***^	0.15^***^	0.73^***^	−0.05
	L1E	−0.17^***^		0.1^**^	0.11^***^	0.76^***^	−0.08^**^
	L2E	−0.08		0.17^***^	0.15^***^	0.75^***^	−0.04
Haptic	L1K	−0.29^***^	0.17^***^		0.04	0.22^***^	0.37^***^
	L1E	−0.41^***^	0.1^**^		−0.11^***^	0.17^***^	0.35^***^
	L2E	−0.32^***^	0.17^***^		−0.04	0.19^***^	0.34^***^
Interoceptive	L1K	0.12^***^	0.15^***^	0.04		0.1^**^	−0.34^***^
	L1E	0.07	0.11^***^	−0.11^***^		0.06	−0.51^***^
	L2E	0.11^***^	0.15^***^	−0.04		0.15^***^	−0.42^***^
Olfactory	L1K	−0.02	0.73^***^	0.22^***^	0.1^**^		0.06
	L1E	−0.19^***^	0.76^***^	0.17^***^	0.06		0.03
	L2E	−0.07	0.75^***^	0.19^***^	0.15^***^		0.03
Visual	L1K	−0.32^***^	−0.05	0.37^***^	−0.34^***^	0.06	
	L1E	−0.36^***^	−0.08^**^	0.35^***^	−0.51^***^	0.03	
	L2E	−0.31^***^	−0.04	0.34^***^	−0.42^***^	0.03	

^*^p < 0.05, ^**^p < 0.01, ^***^p < 0.001.

Orange shade indicates negative correlation and blue positive, with darker color being stronger correlation.

In addition to this, there were other weak significant correlations. There was a weak correlation between interoception and gustatory, between haptic and olfactory, and between haptic and gustatory for all language groups, which were consistent with previous studies (Connell and Lynott, 2012; Vergallito et al., 2020). Since all of these senses had low strengths on average (many of words were rated as near-zero for these senses, particularly for olfactory and gustatory), these weak correlations might be driven by the relationship between only several words.

Some of significant correlation were inconsistent across language groups. Auditory sense had a weak negative correlation with gustatory and olfactory only in L1 English group. The negative correlations between them were also found from English speakers (Connell and Lynott, 2012) and Italian speakers (Vergallito et al., 2020), but not from Korean speakers and L2 English learners in this study. Intuitively, something that can be tasted or smelled is unlikely to be experienced through auditory sense, so it is plausible to find negative correlation between them. However, it is also not surprising that a negative correlation does not exist since they may have no correlation with each other. Moreover, since gustatory and olfactory were evaluated as near-zero in many words, it was possible that a few words changed overall strength of the correlation. Other weak positive or negative correlations (Interceptive and Auditory, Haptic and Interoceptive, Olfactory and interoceptive) were found in only one or two language groups. Since they were also not robust, it was unlikely that they reflected a significant cross-linguistic differences.

### 3.6. Relationship between modalities and concreteness

The correlation between perceptual strengths and concreteness were examined to see whether concreteness reflected or summarized the perceptual properties of the words. Since the survey on concreteness ratings were not conducted for each language group, especially in Korean, this analysis should only be regarded as exploring trends. Concreteness rating had a weak negative correlation with audio and a moderate negative correlation with interoception, while having a weak correlation with olfactory, a moderate positive correlation with haptic and a strong correlation with vision (Table 5). Negative correlation with audio and positive correlation with vision, touch and smell were also reported in previous studies (Connell and Lynott, 2012; Vergallito et al., 2020). The result also seemed to be congruent with our intuition in that something visible or touchable is generally considered more concrete. Those with high visual and haptic strength are more visible and touchable, thus more concrete, while those with high auditory and interoceptive are not, so more abstract. Olfactory words are not always visible and touchable but may have some concreteness in them since experience through smell often allows imagining a physical entity related to it.

**Table 5:** The correlation matrix between *concreteness* and each modality, max strength, and exclusivity.

		Aud.	Gus.	Hap.	Int.	Olf.	Vis.	Max.	Excl.
Conc.	**L1K**	−0.21^***^	0.01	0.33^***^	−0.45^***^	0.11^***^	0.48^***^	0.5^***^	0.24^***^
	**L1E**	−0.23^***^	0.09^**^	0.51^***^	−0.41^***^	0.21^***^	0.54^***^	0.57^***^	0.13^***^
	**L2E**	−0.15^***^	0.05	0.41^***^	−0.49^***^	0.13^***^	0.57^***^	0.55^***^	0.23^***^

^*^p < 0.05, ^**^p < 0.01, ^***^p < 0.001, Aud. = Auditory, Gus. = Gustatory, Hap. = Haptic, Int. = Interoceptive, Olf. = Olfactory, Vis. = Visual, Max. = Max Strength, Excl. = Exclusivity, Conc. = Concreteness.

Orange shade indicates negative correlation and blue positive, with darker color being stronger correlation.

In addition to the correlation between concreteness and six modalities, the correlation with max strength and exclusivity was also analyzed. Max strength is the highest score among perceptual strengths of six modalities, which was one of the best predictors for the response times and accuracies of lexical decision tasks (Connell and Lynott, 2012; Lynott et al., 2020). Max strength was strongly correlated with concreteness. This result was expected because more than 80% of the words were visually dominant, the exclusivity of the visual words was high, and visual strengths had a strong correlation with concreteness. In fact, visual and max strength also showed a strong correlation with each other. Exclusivity and concreteness showed a weak correlation. In other words, the less dimensional the word was, the more concrete. Given that words with high exclusivity were mostly visual words and those with low exclusivity were gustatory or interoceptive, this also seemed to be derived from the correlations between vision and concreteness.

### 3.7. Individual cases

#### 3.7.1. Thermal/hard

**Figure 2:**
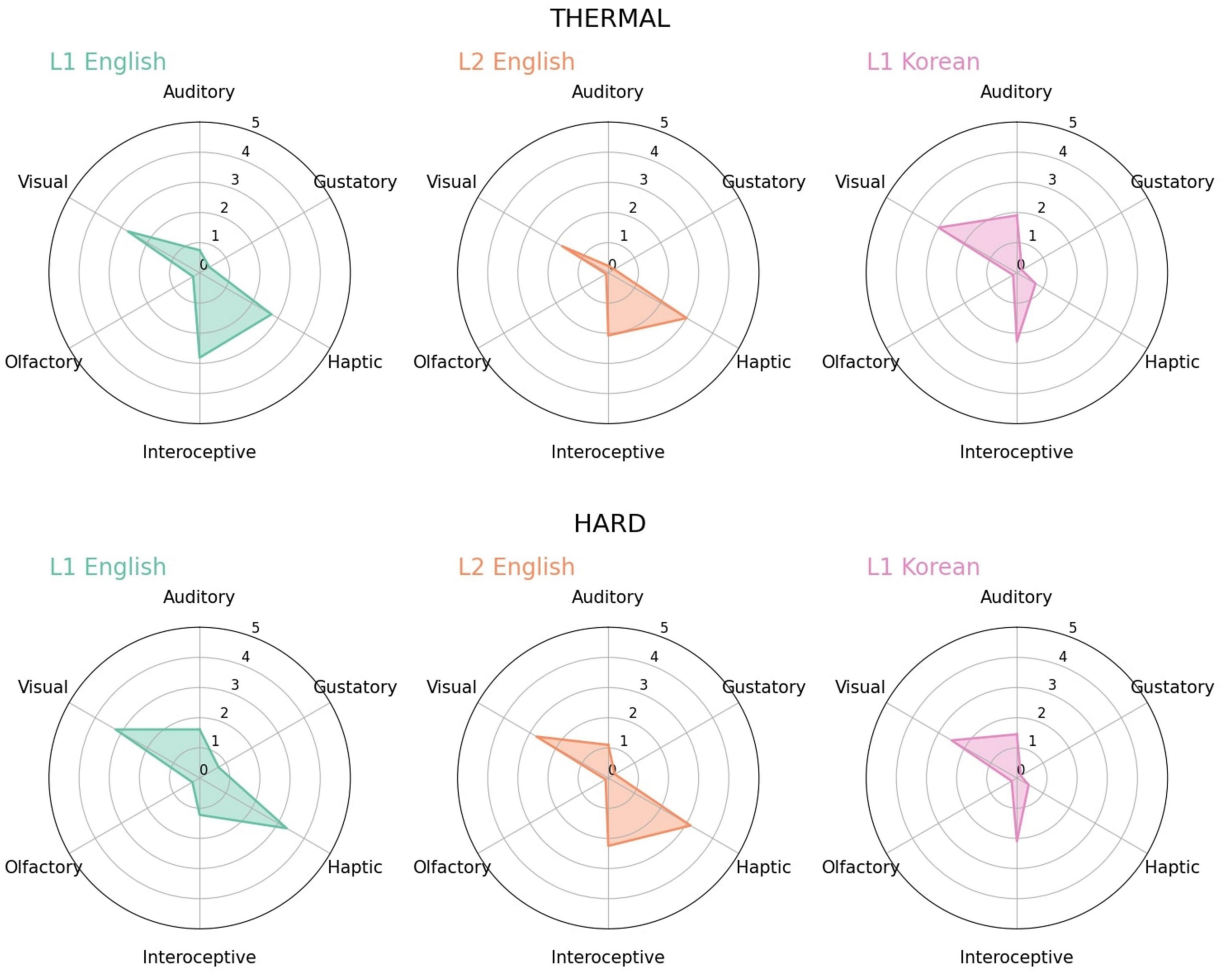
Radar charts for *thermal* (‘yel-uy’) and *hard* (‘elyewun’) in L1 English, L2 English and L1 Korean.

When there was a difference in meaning and sensory experiences related to it between languages, the difference seemed to be sensitively reflected in perceptual strengths. *Thermal* was regarded as a word related to haptic and interoceptive modality in both L1 English and L2 English, while the haptic strength was evaluated relatively low in L1 Korean (Figure 2-top). This difference seemed to be because the Korean translation of thermal (‘yel-uy’) has also a different meaning, “enthusiasm,” which was less likely to be experienced through haptic sense. Given the low haptic strength, Korean participants seemed to generally understand this word as the meaning of enthusiasm. The example of *hard* (Figure 2-bottom) was a similar example with this, but in this case, English is a polysemy and one of the definitions, “difficult,” was selected as the Korean translation (‘elyewun’). In Korean, as the meaning of “solid” was lost, the strength of haptic was lower than in other groups.

#### 3.7.2. Calm/feel

**Figure 3:**
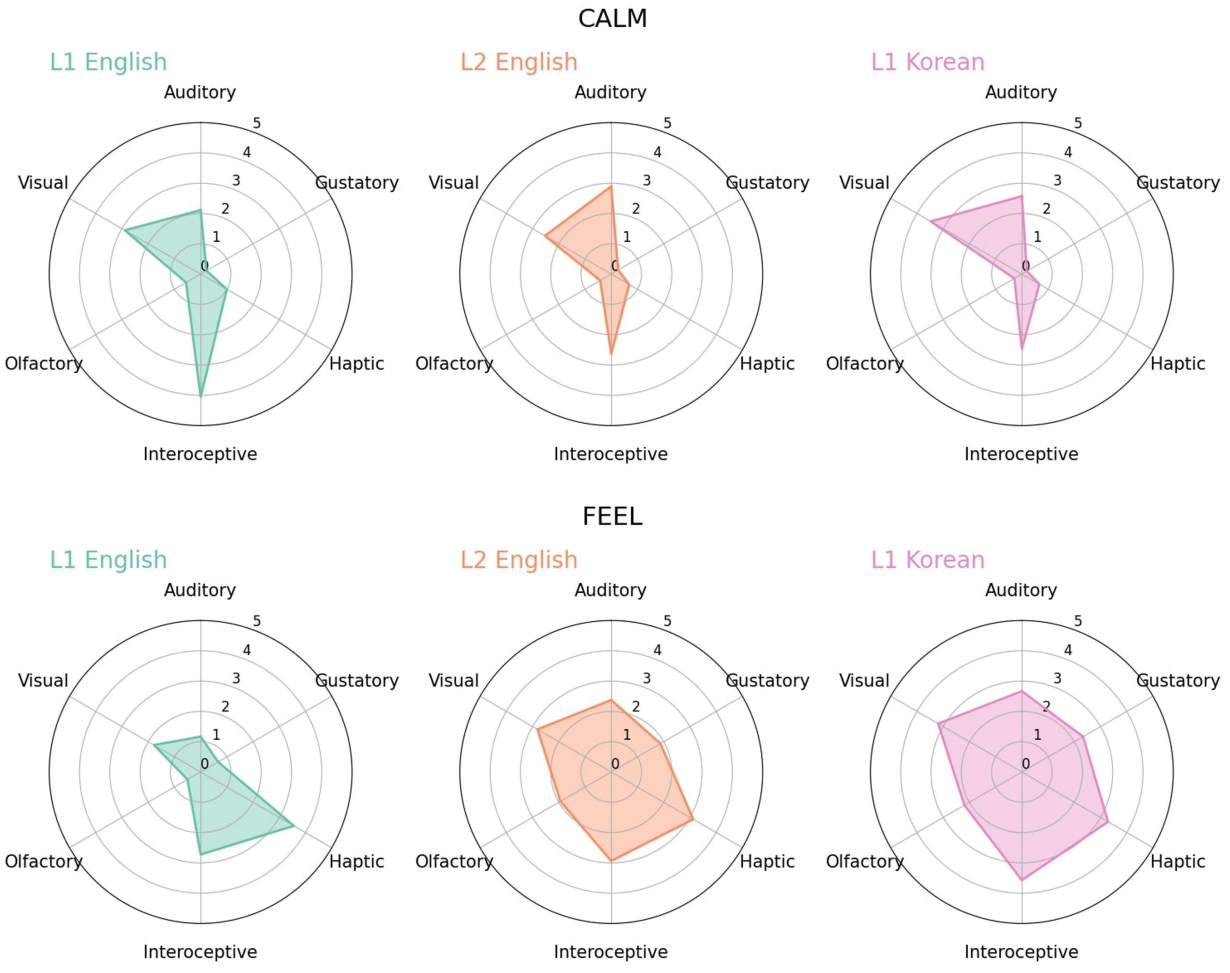
Radar charts for *calm* (‘chapwunhan’) and *feel* (‘nukkita’) in L1 English, L2 English and L1 Korean. In *feel*, compared to L1 English, L2 English and L1 Korean have a similar shape and occupy a wider area.

Some examples showed that, even when the difference in meaning between languages was more nuanced compared to that of polysemy, such subtleties could be captured by language users with the evaluation of perceptual strength. One such example was *calm* (Figure 3-top). *Calm* was interoception-dominant word in L1 English (interoception: 4.06, audio: 2.11, vision: 2.89), while audio-dominant in L2 English (interoception: 2.63, audio: 2.89, vision: 2.53) and vision-dominant in L1 Korean (interoception: 2.48, audio: 2.57, vision: 3.48). L1 English participants seemed to focus more on the (undisturbed, peaceful) internal state, while L2 English participants more on the (quiet) external environment, suggesting that L2 participants might have a different understanding or experience of calm. Meanwhile, when *calm* was translated into a Korean word (‘chapwunhan’), other scores being similar, the dominance of vision increased. Although in Korean, the basic dictionary definition of *clam* is related with one’s inner state, it is in fact frequently used to describe one’s appearance such as in ‘calm hairstyle.’ In other words, a Korean translation of *calm* has a different implication and this subtle difference was well captured in perceptual strengths and dominant modality.

An example of *feel* (Figure 3-bottom) was another case of subtle semantic differences between languages. In addition, it showed that L2 English participants evaluated perceptual strengths under the influence of their native language, Korean. For *feel*, haptic was the dominant modality in L1 English and L2 English, but in L2 English, the perceptual strength for other senses, especially, interoceptive was as high as tactile (haptic: 3.13, interoceptive: 2.94). This might be derived from the influence of Korean. A Korean translation equivalent of *feel* (‘nukkita’) is defined as recognition through all sensory organs rather than focusing on haptic, and is highly likely to cooccur with the experience of emotion, which is known to be relevant with interoception. In fact, *feel* was an interoception dominant word and its strength was 3.57, in L1 Korean. On the other hand, in English, *feel* has a similar meaning as well, but ‘touching by fingers’ plays an important part in definition of *feel*, which might lead to larger exclusivity on haptic. The distribution of L2 English on *feel* appeared to be somewhere in the middle between L1 Korean and L1 English. Individual cases such as *feel* suggested that, despite the overall similar distribution between languages, for certain words, the sensory experience of words might be different depending on its L1 background or usage of words.

#### 3.7.3. Bible

**Figure 4:**
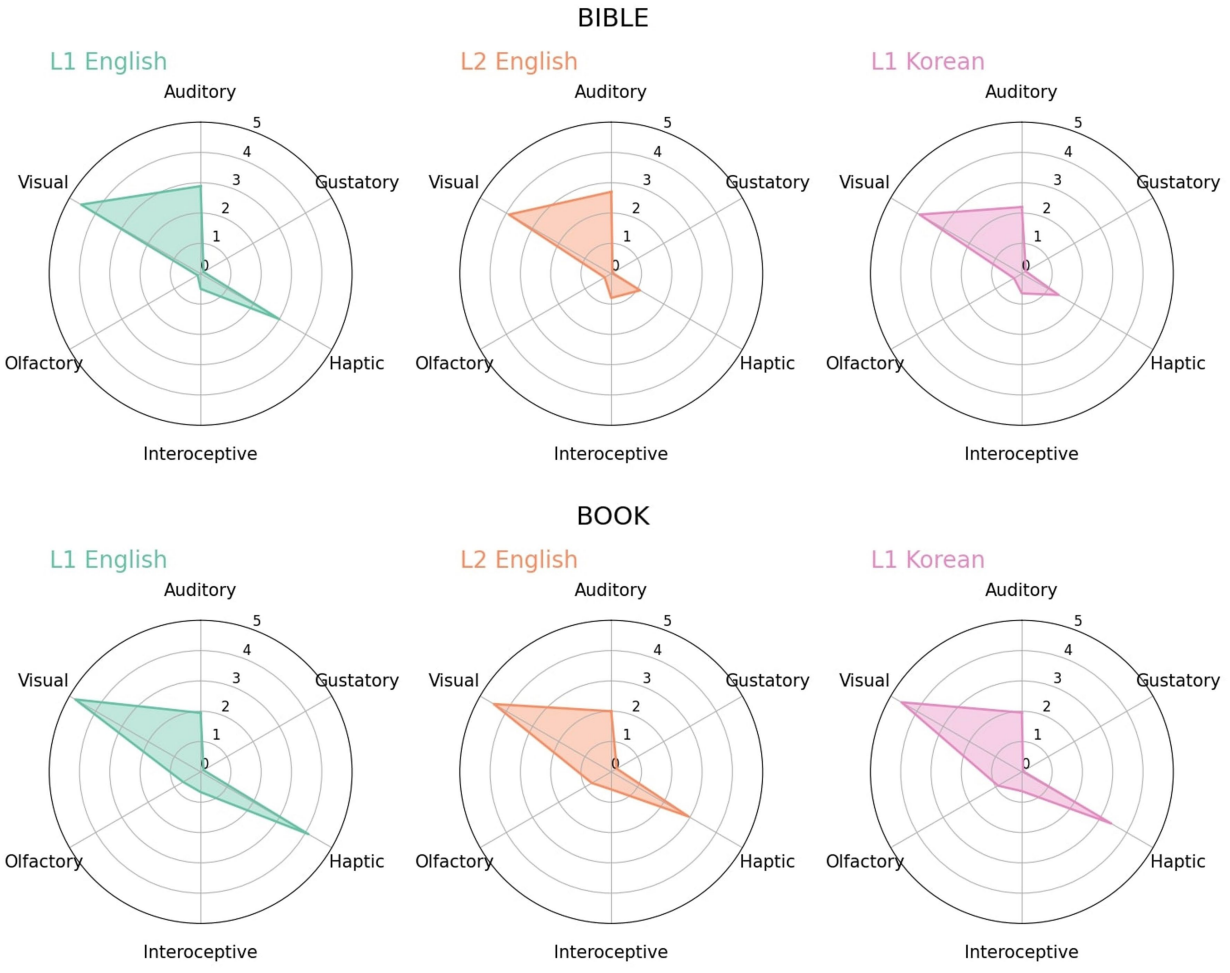
Radar charts for *bible* and *book* in L1 English, L2 English and L1 Korean. For *bible*, the haptic strength of L2 English and L1 Korean is about 1, but it is 3 or higher in *book*.

*Bible* was the case that, as with *feel*, subtle differences in word meaning were reflected in perceptual strengths and the distribution of L2 English seemed to be influenced by Korean. However, it was different from the previous examples in that it was more related to the language users’ experience on the environment surrounding words. *Bible* (Figure 4-top) has a conceptual or symbolic meaning, but it is also a tangible object that actually has a referent in the real world. If its meaning as an object is highlighted more, its visual or haptic properties will be more emphasized. Visual strength was scored as 4 in all three language groups. This may imply that they all regarded bible as an object, although it may possibly reflect the visual imagination of biblical figures. However, haptic strength was rated as 3 or higher only in L1 English and relatively low in the other two groups. L1 English speakers probably had more direct and indirect experiences of actually touching the Bible, so they viewed it as something touchable. For instance, they may have been more strongly influenced by the Christian culture and have had more experience of reading the pages of the Bible or touching it for testimony. On the other hand, L1 Korean and L2 English participants perceived it as a visible, but they associated it less with haptic senses possibly because they had little experience actually touching it.

As for *book* (Figure 4-bottom), a hypernym of *bible* as an object, participants of all language groups rated haptic strength strongly, although L1 English still had the highest among the languages. Therefore, it was not that L2 English and L1 Korean participants considered a book-like item itself as unable to be experienced with haptic sense. It appeared that they regarded the Bible as rather distinct from books or had little experience touching or reading it with hand. Thus, the case of *bible* demonstrated that the perceptual strengths delicately represented the sensory experience of words and that the evaluation of L2 English participants was similar to that of L1 Korean participants with the same sensory experience.

### 3.8. Individual cases for loanwords

Loanwords are words borrowed from a different language (source language) and adopted by the speakers of the target language. Because the word and its referent are borrowed together, loanwords correspond with the original counterparts on the surface. However, since only a part of meanings is borrowed in the process of borrowing or the context of usage might vary, the two words often do not match completely. Analyzing these words might provide interesting examples in the comparison of perceptual strengths between languages. On the surface, English and Korean translations correspond, so in most cases the same perceptual strengths will be observed in Korean and English. However, depending on the context or usage, they may differ between Korean and English, and for those cases, it is noteworthy examining how L2 English participants’ evaluations are influenced. Despite English scripts, the words share pronunciation with Korean, so L2 English participants may perceive them to be Korean words and evaluate them similarly in Korean. Otherwise, the influence of English transcription is so strong that participants may accept the words as English rather than Korean and rate them more similarly to L1 English. This section focuses on the loanwords and offers several individual examples.

#### 3.8.1. Cake

**Figure 5:**
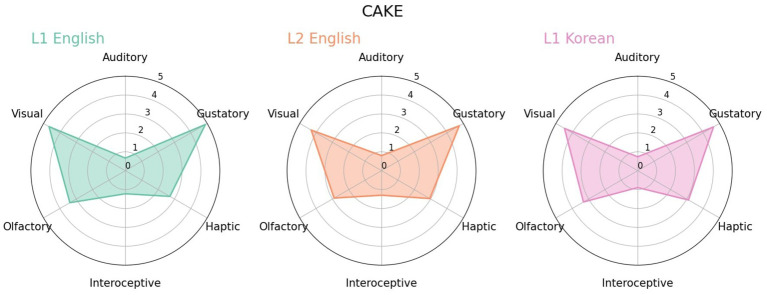
Radar charts for *cake* in L1 English, L2 English and L1 Korean.

For the vast majority of loanwords, the strengths and distribution of the three groups were similar. Typical examples were food-related terms, such as *cake* (Figure 5). Since they refer to the same object and the sensory experience for them would be similar, the evaluations of the three language groups were nearly identical.

#### 3.8.2. Audio/blues/musical

**Figure 6:**
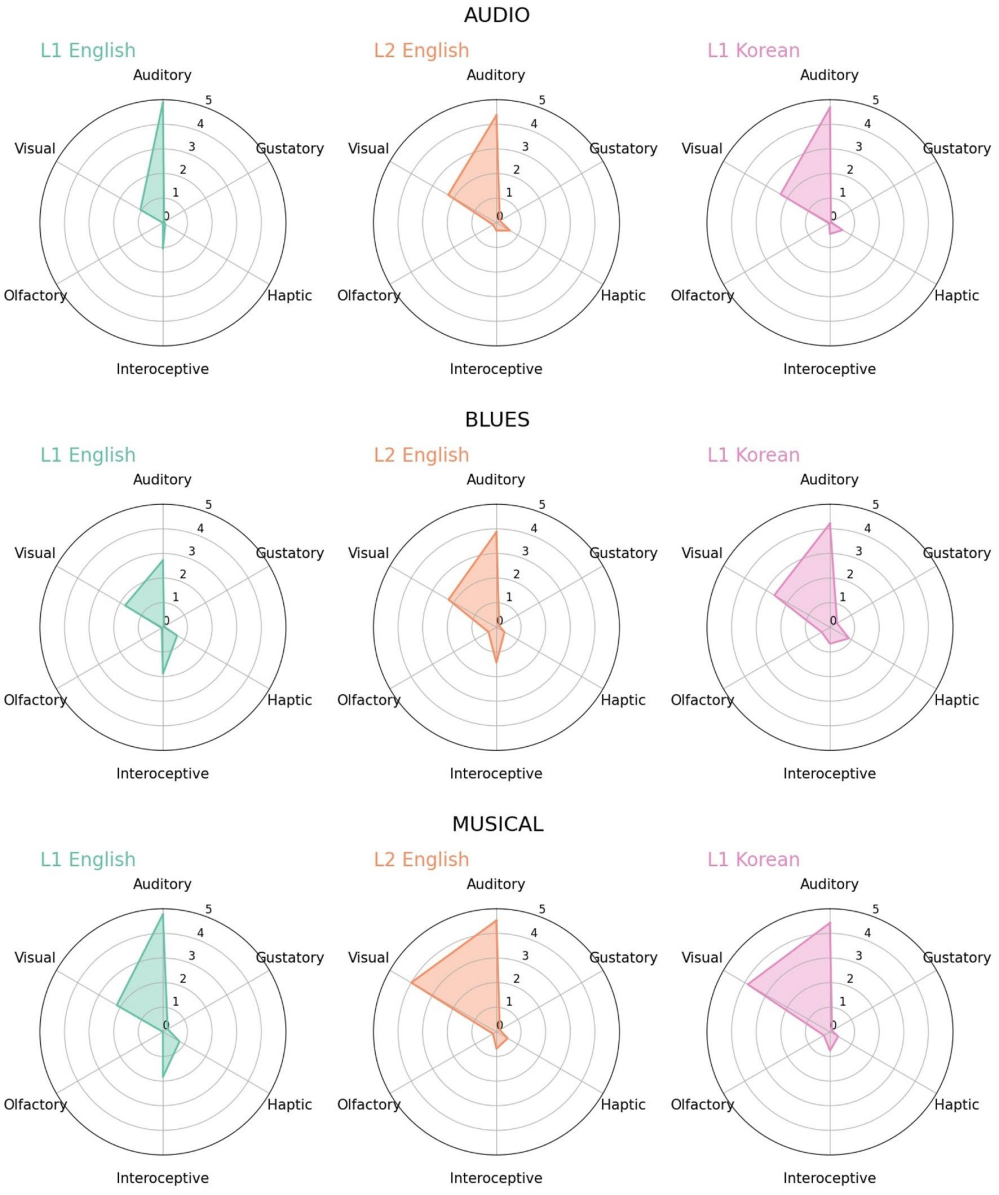
Radar charts for *audio*, *blues* and *musical* in L1 English, L2 English and L1 Korean.

L2 English participants rated their perceptual strengths similarly to L1 Korean rather than L1 English in several words. Examples were *audio*, *blues* and *musical*. Of these, *audio* (Figure 6-top) was a case where Korean loanwords had additional meaning than English. For audio and its loanword (‘wodio’), visual strengths were evaluated as 2 or higher both in L2 English and L1 Korean. Since the Korean loanword also refers to an audio device, there was a possibility that *audio* could also be recognized as vision from the experience of the device. In contrast, in English, *audio* seldom refers to an audio device, at least by dictionary definition, so it was difficult for L1 English participants to experience it through vision. L2 English participants assessed it more similarly to Korean, revealing the influence from Korean.

*Blues* and *musical* (Figure 6-middle, bottom) were cases in which only part of the English word’s meanings is borrowed. *Blues* refers to a genre of music or feelings of melancholy in English, while the Korean loanword (‘pulusu’) is mostly used in the former meaning and has been extended to include the meaning of dancing to such music. In English, along with auditory strength, interoception was evaluated relatively high [3 or higher in Lynott et al. (2020)], which is known to be related with emotion. On the other hand, in Korean, the word was evaluated as having relatively higher auditory and visual strength but lower interoception, reflecting that in Korean, *blues* is mainly associated only with music or dance as in the dictionary definition. The distribution of L2 English was not completely different from that of L1 English, but largely overlapped with that of L1 Korean.

*Musical* refers to an adjective of music and a type of the performance genres in English, whereas it is borrowed only as the latter meaning in Korean. Therefore, both audio and visual strength were high in Korean, seemingly reflecting the experience of musical performance. On the other hand, the evaluation of L1 English seemed to take into account both meanings, but prioritizing the former, as shown in high auditory strength and relatively lower visual strength. The distribution of L2 English was almost identical to that of Korean, not L1 English. All of these cases demonstrate that L2 English participants recognized Korean loanwords as they would in their native language rather than English, despite the different orthographical from.

## 4. Discussion

The present study established the perceptual strengths norms of L1 Korean, L1 English, and L2 English for future research (The norms are freely available from the link in *Data Availability Statement*), and explored how human sensory experiences affect semantic processing and how these were different across languages and between L1 and L2. The norms of each language showed reliable results compatible with human experience and consistent within raters and demonstrated that three language groups generally had a similar distribution in perceptual strengths despite a few differences in individual words. Further discussions of the results will be provided below, focusing on the comparison between languages, the relationship between modalities, and the relationship between concreteness and perceptual strengths.

Overall, no significant differences were found in the distribution of perceptual strengths between L1 Korean, L1 English, and L2 English. The correlation coefficients between languages were significantly large and the Euclidean distances between languages were relatively small. In other words, the similarity between languages was large. For all three language groups, visual strength was the highest, followed by auditory, haptic, interoceptive, olfactory, and gustatory. Dominant modalities also had the highest proportion of vision, accounting for almost 80% or more in all languages. However, the ranking of the most dominant modality, where vision was followed by auditory, interoceptive, gustatory, haptic and olfactory, was different from that of perceptual strength mean. Words with greater exclusivity were all visual words in common, and less exclusive words were generally those with high gustatory strength. It was not surprising that three groups had similar distributions, since each word used in the study referred to the same referent at least on the surface level and there was no reason to assume the environments surrounding the languages were so different from each other. In fact, the distributions in this study were also similarly observed in surveys of other languages (Lynott et al., 2020; Vergallito et al., 2020; Miceli et al., 2021; Speed and Brybaert, 2022), even though they slightly differed depending on which words were investigated—for instance, haptic strength was higher than auditory in Miceli et al. (2021), where most words were concrete nouns. However, since there was no research conducted on Korean and L2 English and no direct cross-linguistic comparison on the identical word list, this comparison between L1 Korean, L1 English and L2 English provided more direct evidence for the similarity of the distribution in perceptual strengths across languages. In particular, the finding that L1 English and L2 English showed a generally similar distribution was of significance in researching L2 embodiment processing. Several studies have demonstrated that L2 processing is also embodied but their research area has been largely limited to action verbs. This study showed that various sensory perceptions other than motor action could be experienced in a similar way and degree to that of L1. Although this was not direct evidence of L2 embodiment, it held that at least L2 learners were able to associate words with the perceptual experience.

The three language groups showed a similar distribution in general, but there were some differences between them. The perceptual strengths of haptic and auditory modalities differed between languages. For both modalities, significant differences were found between L1 English and both L1 Korean and L2 English. For dominant modalities, L1 Korean had a greater proportion of vision and smaller proportion of interoceptive and haptic modalities than the other two language groups. For exclusivity, some of the least exclusive words in L2 English and L1 Korean were interoceptive-dominant, but all were gustatory words in L1 English. However, these findings warrant a careful interpretation, given several key considerations. Firstly, despite statistical differences in certain aspects across the languages, the overall patterns were not markedly distinct. For example, although the interoceptive or haptic dominant words in the other languages were labeled as visual words in L1 Korean, their interoceptive and haptic strengths were greater than average. As suggested in Speed and Brybaert (2022) there can be potential variances even among similar participant groups, the observed differences among our language groups may not be definitive. Secondly, since Korean and English do not have an exact one-to-one correspondence, it is difficult to interpret the difference between Korean and English simply as caused by linguistic difference in sensory experiences. Not only polysemy or homonym but also any word translated into another language might produce subtle differences in lexical meaning from the original. For this reason, the translation-equivalent word list itself might reflect the cultural differences. Finally, demographic disparities, specifically age differences among the language groups, demand consideration. The L1 English group presented greater heterogeneity and an older average age compared to the L1 Korean and L2 English groups, potentially influencing language acquisition and use, as well as cognitive functioning (Craik and Bialystok, 2006), which might contribute to the observed differences. Therefore, it would be more valuable to compare overall trends and distributions rather than highlight a few differences in perceptual strengths or exclusivity between languages.

Although comparisons across languages based on differences in average strengths or exclusivity were often difficult to interpret due to diverse factors mentioned above, differences in individual words might be able to provide some insights on how differences between languages were reflected in perceptual strengths. The examination of specific cases such as *feel* and *calm* demonstrated that the perceptual strengths of L1 Korean and L1 English captured with great sensitivity the variation in meaning between English word and its Korean translation. For instance, the Korean translation of *feel* relates to emotions and a broader range of senses than its English counterpart, which was revealed in the high interoceptive strength and low exclusivity in the Korean rating. When there was no one-to-one relationship between the English and Korean translations for a particular word, the rating of the word became notably dissimilar. For example, if an English polysemy loses some of its meanings when translated into Korean, only the perception of one meaning was evaluated in Korean (e.g., *hard* was not rated as haptic in Korean because it is translated into “difficult”). This sensitivity illustrated that participants did not judge the perceptual strength of language randomly, but rather in accordance with genuine language perception. Obviously, this sensitivity, which led to an intriguing analysis, is also a characteristic that makes cross-language research more challenging. It is imperative to exercise caution in the selection of items when conducting comparisons across languages.

Another important question of the present study was whether the L2 English participants’ ratings showed a distribution more similar to English or Korean. In the overall trend, L2 English appeared to be closer to L1 Korean than L1 English, since the mean of the Euclidean distance between L2 English and L1 Korean was shorter than between L2 English and L1 English. Moreover, the results of the individual cases indicated that in instances where the distributions between L1 English and L1 Korean were distinct, L2 English tended to be more similar to that of L1 Korean. For example, the judgements made by L2 English participants regarding the word, *feel*, were found to be more comparable to those of L1 Korean participants than L1 English participants. This was evidenced by the radar chart (Figure 3), which illustrated that the form of the graph for L2 English was more similar to that of L1 Korean, and the Euclidean distance between the two was closer (L1 English vs. L2 English: 2.51, L1 Korean vs. L2 English: 0.96). Comparisons of loanwords also revealed that when there were differences between Korean and English, the distribution of L2 English evaluations was more likely to be comparable to that of L1 Korean, such as in *audio* and *musical*. These findings suggested that although L2 participants evaluated perceptual strengths through English, their assessments were rather similar to those of Korean participants with similar cultural and linguistic backgrounds.

The closer alignment of L2 English with L1 Korean may imply that the semantic representations of L2 speakers are significantly influenced by their native language, even when engaging with their second language. This is in line with Jiang’s (2000) model of vocabulary acquisition, which postulates that L2 vocabulary acquisition progresses through stages that necessitate the activation and mediation of L1 semantic information (See also, Jiang, 2002). The initial and intermediate stages involve the lexical association stage, where L2 word usage triggers associations with L1 translation equivalents, and the L1 lemma mediation stage, where semantic information is transferred from L1 to L2. In each of these stages, both direct and indirect utilization of L1 semantic information facilitates L2 word semantic processing. Hence, these theories predict that the perceptual evaluations of L2 words by L2 speakers will manifest in a manner similar to their L1. Our results further align with the predictions of the Modified Hierarchical Model (Pavlenko, 2009) for bilingual lexicon models. This model posits that the L1 and L2 lexicons are interconnected and both of them are, respectively, connected to the three conceptual elements—shared, L1-specific, and L2-specific elements. The model highlights that the L1 or shared concepts are employed prior to the L2-specific elements, particularly when the learner’s proficiency with a certain word is not sufficiently advanced. Accordingly, in the context of our study, we can observe that the L2 participants initially leveraged their L1 semantic representations in the evaluation of perceptual strength for certain words. Considering our findings alongside the predictions of the models, we can infer the potential underlying representations and processes when L2 learners encounter L2 words. First, upon encountering an L2 word, they may initially activate the corresponding L1 translation, comprehending the word *via* lexical mediation. Alternatively, they may utilize L2 words with their semantic representation copied from L1 representations. Or it could be the case that they have learned the L2 word in an environment distinct from that of native speakers of the target language, leading them to perceive the word similarly to their native language. It is not feasible to exclusively support one of these possibilities based solely on our findings. However, these scenarios all remain plausible and could be influenced by various factors, such as the language development process or proficiency level.

The relationship between modalities did not differ significantly between languages. Although there seemed to be some differences (for example, negative correlation between auditory and gustatory or olfactory is only found in L1 English), since the majority of the differences were weak correlations and all languages exhibited a generally similar pattern, it was difficult to conclude that the differences were due to language differences. The correlation between modalities was congruent with the intuition on human perception. A positive correlation between olfactory and gustatory, positive correlation between visual and haptic, negative correlation between auditory and visual, and negative correlation between interoceptive and visual were all consistent with our understanding of human perception and they were also observed in previous studies. As indicated by the excellent inter-raters’ consistency, the raters’ assessments were not arbitrary but consistent among themselves and this well-agreed evaluation seemed to represent the experience of human perception. This demonstrated that it was possible for language users to plausibly reproduce the experience of the world with language as a cue; that is, language and the perceptual experience of the associated referent interact with each other.

One of the observed correlations worth noting was the negative correlation between interoception and vision. Because interoception has been rarely explored in perceptual strengths as one of modalities; hence, investigations on their correlation with other modalities were also uncommon (Lynott et al., 2020; Miceli et al., 2021). This study confirmed, consistent with earlier research, that interoception was negatively correlated with vision in Korean and L2 English as well. The finding that interoception was negatively correlated with vision suggested that interoception should be addressed in perceptual strength research alongside the five senses. Interoception can compensate for the aspects of the world that cannot be explained by vision, the sense that accounts for the largest portion of the sensory modalities. When perceptual strength is assessed without interoception, a word with weak visual strength may be deemed incomprehensible by sensory experience.

The importance of interoception was also revealed in the relationship between concreteness and modalities. Concreteness had a positive correlation with visual, haptic, and olfactory, and a negative correlation with interoception and audio in this study. Interoception was only modality having a modest negative correlation with concreteness. If the sensory experience of abstract words was evaluated without interoception, some of them would be rated as having low perceptual strength in any sense and would be considered as words that could not be experienced *via* the sensory modalities. Although abstract words were often evaluated as being related to auditory modality, audio alone was not able to describe all of the sensory experiences associated with abstract concepts. Evaluating abstracts words not having any sensory experience may lead to the erroneous conclusion that the embodiment of some of abstract concepts cannot be explained with the sensory-motor domain. Therefore, constructing perceptual strength norms including interoception is necessary to further explore abstract words in terms of embodiment cognition.

It was also worth noting that the correlations between concreteness and each modality varied. Some were negatively correlated, some were positively correlated, and some were only weakly correlated. In other words, it was difficult to capture all the senses with a single measure of concreteness. Nevertheless, one may argue that it is more effective to employ a single dichotomy, such as concreteness, rather than multiple sensory indices. Furthermore, concreteness showed a high correlation with max strength, which represents the strongest sensory modality for a given concept, indirectly reflecting the influences of all senses. In fact, previous studies have shown that concreteness can explain a variety of linguistic phenomena such as concreteness effect and predict accuracies and response times of lexical decision tasks (James, 1975; Marschark and Paivio, 1977). However, being able to sufficiently explain the several phenomena only with concreteness is likely to be an illusion caused by vision dominance (Connell and Lynott, 2012). Since there is a strong positive correlation between vision and concreteness (Connell and Lynott, 2012; Miceli et al., 2021; Speed and Brybaert, 2022), employing concreteness has the similar effect as utilizing visual perceptual strength (Connell and Lynott, 2012). This visual strength alone, since most words are visually dominant, can explain large proportion of semantic processing, and concreteness take advantage of this. In this respect, the explanatory power of concreteness may be proportional to how many visual words are contained in the item lists. Indeed, the well-known concreteness effect, in which concrete words are processed faster than abstract words, was reversed when emotion-related words, presumably less visual dominant words, were used as materials (Kousta et al., 2011). Therefore, substituting multiple sensory indices with concreteness may appear to be an easy solution, but it is not the most effective way to describe actual conceptualization processing.

## 5. Conclusion

This study constructed perceptual strength norms of 1,000 words for L1 Korean, L1 English, and L2 English. Through it, the similarity and difference between languages were compared and the influence of environment on semantic processing was explored. This was the first study to establish the norm for Korean and L2 English and directly compare languages using the norm with the identical word list across groups. The results showed that perceptual strength norms were not arbitrary but had psycholinguistic reality. Perceptual strengths were highly consistent among the participants and were congruent with human sensory experience. In addition, the evaluations were sensitive enough to reflect subtle variation in languages. Comparisons between languages revealed that all three language groups similarly rated the perceptual strengths of words. This indicated that language users had in general parallel sensory experiences with concepts and that L2 learners as well as L1 speakers were able to associate their sensory experiences with linguistic concepts. The results also revealed that the evaluation of individual words may vary depending on linguistic and cultural experiences. When the perceptual strengths between Korean and English were dissimilar, L2 participants frequently gave ratings similar to those of L1 Korean, implying the L1 and environmental influences on L2 semantic representations. In addition, the present study demonstrated that it was crucial to incorporate interoception as a sensory modality when developing the perceptual strength norm. It had a negative correlation with both vision and concreteness. If perceptual strengths were measured with only the five commonly used senses without interoception, many words would be misled as not being understood through sensory experiences. With the inclusion of interoception, abstract concepts, which appeared to be unexplainable by sensory perception, could be described within the sensory-motor system.

## Data availability statement

The datasets presented in this study can be found in online repositories. The names of the repository/repositories and accession number(s) can be found at: https://github.com/coolmintmild/materialrepository.

## Ethics statement

The studies involving human participants were reviewed and approved by the Ethical Committee of Seoul National University. The patients/participants provided their written informed consent to participate in this study.

## Author contributions

JL and J-AS conceived and designed the study. JL conducted the experiment, analyzed the data, and wrote the manuscript. J-AS reviewed the manuscript. All authors contributed to the article and approved the submitted version.

## Funding

This work was supported by the Park Chung-Jip Scholarship Fund for the Next Generation in English literature and language at Seoul National University in 2022.

## Conflict of interest

The authors declare that the research was conducted in the absence of any commercial or financial relationships that could be construed as a potential conflict of interest.

## Publisher’s note

All claims expressed in this article are solely those of the authors and do not necessarily represent those of their affiliated organizations, or those of the publisher, the editors and the reviewers. Any product that may be evaluated in this article, or claim that may be made by its manufacturer, is not guaranteed or endorsed by the publisher.

## Supplementary material

The Supplementary material for this article can be found online at: https://www.frontiersin.org/articles/10.3389/fpsyg.2023.1188909/full#supplementary-material

## Footnotes

^1^Please note, in response to a reviewer’s concern regarding the potential age-related bias, that the L1 English group was indeed older than the other groups. However, our additional analysis, albeit not reported in the paper, identified only minor/insignificant differences in perceptual strength norms between younger and older subsets within the L1 English group. Therefore, we present data inclusive of all age groups in the L1 English group. Furthermore, to minimize any potential age-related influence, we have incorporated age as a factor in our statistical model.
