# Supplementary material for: The cross-linguistic comparison of perceptual strength norms for Korean, English and L2 English
Source: Front Psychol. 2023 Jul 19;14:1188909. doi: 10.3389/fpsyg.2023.1188909 (PMC10395129; doi:10.3389/fpsyg.2023.1188909)

Supplementary Material

# Wordlist for experiment (English and Korean translation)

* AR: agreement rate among translators

| **English** | **Korean** | **AR** |
| --- | --- | --- |
| ADDICTION | 중독 | 100% |
| AIRPORT | 공항 | 100% |
| ANGRY | 화난 | 100% |
| APARTMENT | 아파트 | 100% |
| ARRIVAL | 도착 | 100% |
| AUCTION | 경매 | 100% |
| BACKGROUND | 배경 | 100% |
| BALL | 공 | 100% |
| BANK | 은행 | 100% |
| BASKET | 바구니 | 100% |
| BLOG | 블로그 | 100% |
| BOOK | 책 | 100% |
| BOTTLE | 병 (용기) | 100% |
| BRICK | 벽돌 | 100% |
| BRIDGE | 다리 | 100% |
| BROAD | 넓은 | 100% |
| BUDGET | 예산 | 100% |
| BUTTER | 버터 | 100% |
| CALENDAR | 달력 | 100% |
| CARD | 카드 | 100% |
| CARTOON | 만화 | 100% |
| CASTLE | 성 (건물) | 100% |
| CATCH | 잡다 | 100% |
| CHAIR | 의자 | 100% |
| CHERRY | 체리 | 100% |
| CITIZEN | 시민 | 100% |
| CLOCK | 시계 | 100% |
| COACH | 코치 | 100% |
| CONTINENT | 대륙 | 100% |
| CRIME | 범죄 | 100% |
| DELICIOUS | 맛있는 | 100% |
| DESK | 책상 | 100% |
| DOLL | 인형 | 100% |
| DOLLAR | 달러 | 100% |
| DRAGON | 용 | 100% |
| EAGLE | 독수리 | 100% |
| EIGHT | 여덟 | 100% |
| ELECTION | 선거 | 100% |
| EVENING | 저녁 | 100% |
| EXAM | 시험 | 100% |
| EXPLAIN | 설명하다 | 100% |
| EXPOSED | 노출된 | 100% |
| FAMOUS | 유명한 | 100% |
| FASHION | 패션 | 100% |
| FINGER | 손가락 | 100% |
| FLOOD | 홍수 | 100% |
| FOLD | 접다 | 100% |
| FOOT | 발 | 100% |
| GIVE | 주다 | 100% |
| GOLF | 골프 | 100% |
| GRAMMAR | 문법 | 100% |
| GRAPH | 그래프 | 100% |
| GRAVITY | 중력 | 100% |
| GRAY | 회색 | 100% |
| GREY | 회색의 | 100% |
| GROWTH | 성장 | 100% |
| HEAR | 듣다 | 100% |
| HERO | 영웅 | 100% |
| HIGHWAY | 고속도로 | 100% |
| HOCKEY | 하키 | 100% |
| HOLE | 구멍 | 100% |
| HONEY | 꿀 | 100% |
| HOUSE | 집 | 100% |
| HUNTER | 사냥꾼 | 100% |
| INCH | 인치 | 100% |
| ISLAND | 섬 | 100% |
| JAPAN | 일본 | 100% |
| JUICE | 주스 | 100% |
| KING | 왕 | 100% |
| KINGDOM | 왕국 | 100% |
| KNIFE | 칼 | 100% |
| KNIGHT | 기사 (말 탄 군인) | 100% |
| LANGUAGE | 언어 | 100% |
| LASER | 레이저 | 100% |
| LEMON | 레몬 | 100% |
| LENGTH | 길이 | 100% |
| LIBRARY | 도서관 | 100% |
| LITERATURE | 문학 | 100% |
| MAGAZINE | 잡지 | 100% |
| MARS | 화성 | 100% |
| MATH | 수학 | 100% |
| MENU | 메뉴 | 100% |
| MIRROR | 거울 | 100% |
| MOVIE | 영화 | 100% |
| NAIL | 손톱 | 100% |
| NARROW | 좁은 | 100% |
| NEIGHBOR | 이웃 | 100% |
| NEST | 둥지 | 100% |
| NEWS | 뉴스 | 100% |
| NIGHTMARE | 악몽 | 100% |
| NUMBER | 숫자 | 100% |
| OLIVE | 올리브 | 100% |
| OLYMPIC | 올림픽 | 100% |
| PARK | 공원 | 100% |
| PASSPORT | 여권 | 100% |
| PEARL | 진주 | 100% |
| PHILOSOPHY | 철학 | 100% |
| PHOTO | 사진 | 100% |
| PIZZA | 피자 | 100% |
| PLANET | 행성 | 100% |
| POEM | 시 | 100% |
| POETRY | 시가 | 100% |
| POLICE | 경찰 | 100% |
| POPULATION | 인구 | 100% |
| PORTFOLIO | 포트폴리오 | 100% |
| PRESIDENT | 대통령 | 100% |
| PRINCE | 왕자 | 100% |
| PRINCESS | 공주 | 100% |
| PULL | 당기다 | 100% |
| RADIO | 라디오 | 100% |
| RAINBOW | 무지개 | 100% |
| RATIO | 비율 | 100% |
| READ | 읽다 | 100% |
| ROOT | 뿌리 | 100% |
| SALT | 소금 | 100% |
| SAND | 모래 | 100% |
| SCIENTIST | 과학자 | 100% |
| SHADOW | 그림자 | 100% |
| SHARK | 상어 | 100% |
| SHOE | 신발 | 100% |
| SHOULDER | 어깨 | 100% |
| SILVER | 은 | 100% |
| SINGER | 가수 | 100% |
| SNAKE | 뱀 | 100% |
| SOCCER | 축구 | 100% |
| SOCIETY | 사회 | 100% |
| SOFTWARE | 소프트웨어 | 100% |
| SPEAK | 말하다 | 100% |
| SPIDER | 거미 | 100% |
| STAGE | 무대 | 100% |
| STAR | 별 | 100% |
| SUGAR | 설탕 | 100% |
| SURFACE | 표면 | 100% |
| TEAM | 팀 | 100% |
| TENNIS | 테니스 | 100% |
| THINK | 생각하다 | 100% |
| THREE | 셋 | 100% |
| THROW | 던지다 | 100% |
| TITLE | 제목 | 100% |
| TROPHY | 트로피 | 100% |
| TUNNEL | 터널 | 100% |
| TWIN | 쌍둥이 | 100% |
| VILLAGE | 마을 | 100% |
| VIRUS | 바이러스 | 100% |
| VOICE | 목소리 | 100% |
| WALLET | 지갑 | 100% |
| WHEEL | 바퀴 | 100% |
| WINDOW | 창문 | 100% |
| WORD | 단어 | 100% |
| ANIMATION | 애니메이션 | 91% |
| APPLE | 사과 (과일) | 91% |
| ARROW | 화살 | 91% |
| BATTERY | 배터리 | 91% |
| BELIEF | 믿음 | 91% |
| BRIDE | 신부 (결혼할 여성) | 91% |
| BUBBLE | 거품 | 91% |
| BUTTON | 버튼 | 91% |
| CHEST | 가슴 | 91% |
| CLIENT | 고객 | 91% |
| CLIFF | 절벽 | 91% |
| CLOUD | 구름 | 91% |
| CONTRACT | 계약 | 91% |
| COPPER | 구리 | 91% |
| CUTE | 귀여운 | 91% |
| DECLARE | 선언하다 | 91% |
| DEER | 사슴 | 91% |
| DESIGN | 디자인 | 91% |
| DIGITAL | 디지털 | 91% |
| DIRECTION | 방향 | 91% |
| DISABILITY | 장애 | 91% |
| DISAPPEAR | 사라지다 | 91% |
| DOCTOR | 의사 | 91% |
| DUST | 먼지 | 91% |
| EMAIL | 이메일 | 91% |
| ENERGY | 에너지 | 91% |
| ENTRANCE | 입구 | 91% |
| EURO | 유로 | 91% |
| FACE | 얼굴 | 91% |
| FILE | 파일 | 91% |
| FLAG | 깃발 | 91% |
| GENTLEMAN | 신사 | 91% |
| GIFT | 선물 | 91% |
| GRADUATION | 졸업 | 91% |
| HEALTH | 건강 | 91% |
| HILL | 언덕 | 91% |
| HOMEWORK | 숙제 | 91% |
| HORN | 뿔 | 91% |
| HOTEL | 호텔 | 91% |
| INDUSTRY | 산업 | 91% |
| INTERNET | 인터넷 | 91% |
| LAND | 땅 | 91% |
| LATE | 늦은 | 91% |
| LEADERSHIP | 리더십 | 91% |
| LENS | 렌즈 | 91% |
| LIGHTNING | 번개 | 91% |
| LOCKED | 잠긴 | 91% |
| LONELY | 외로운 | 91% |
| LOOK | 보다 | 91% |
| LYRICS | 가사 | 91% |
| MAYOR | 시장 | 91% |
| MEDAL | 메달 | 91% |
| MISSILE | 미사일 | 91% |
| MUSIC | 음악 | 91% |
| NEWSPAPER | 신문 | 91% |
| NIGHT | 밤 | 91% |
| NURSE | 간호사 | 91% |
| OPEN | 열다 | 91% |
| OXYGEN | 산소 | 91% |
| PANTS | 바지 | 91% |
| PASSWORD | 비밀번호 | 91% |
| PEACEFUL | 평화로운 | 91% |
| POCKET | 주머니 | 91% |
| POSTER | 포스터 | 91% |
| PRICE | 가격 | 91% |
| PROFESSOR | 교수 | 91% |
| PROGRAM | 프로그램 | 91% |
| QUESTION | 질문 | 91% |
| RUDE | 무례한 | 91% |
| SALAD | 샐러드 | 91% |
| SEEK | 찾다 | 91% |
| SMALL | 작은 | 91% |
| SOLAR | 태양의 | 91% |
| SOLDIER | 군인 | 91% |
| SOUND | 소리 | 91% |
| STORY | 이야기 | 91% |
| STRESS | 스트레스 | 91% |
| STRUCTURE | 구조 | 91% |
| STUDIO | 스튜디오 | 91% |
| STYLE | 스타일 | 91% |
| SUGGESTION | 제안 | 91% |
| TAIL | 꼬리 | 91% |
| TALE | 설화 | 91% |
| TAPE | 테이프 | 91% |
| TEACHER | 선생님 | 91% |
| TERRORIST | 테러리스트 | 91% |
| THUNDER | 천둥 | 91% |
| TOOL | 도구 | 91% |
| UGLY | 못생긴 | 91% |
| VISA | 비자 | 91% |
| VISIT | 방문하다 | 91% |
| WISDOM | 지혜 | 91% |
| ADDRESS | 주소 | 82% |
| AIRLINE | 항공사 | 82% |
| ALARM | 알람 | 82% |
| ANCIENT | 고대의 | 82% |
| ANGLE | 각도 | 82% |
| APPEAR | 나타나다 | 82% |
| ARMED | 무장한 | 82% |
| BEHAVIOR | 행동 | 82% |
| BIBLE | 성경 | 82% |
| BRIGHT | 밝은 | 82% |
| BRONZE | 동 | 82% |
| BROWN | 갈색 | 82% |
| CAKE | 케이크 | 82% |
| CASH | 현금 | 82% |
| CLASSROOM | 교실 | 82% |
| CLOSET | 옷장 | 82% |
| COAL | 석탄 | 82% |
| COAT | 코트 | 82% |
| COIN | 동전 | 82% |
| COLOR | 색깔 | 82% |
| CONFIDENCE | 자신감 | 82% |
| CROWN | 왕관 | 82% |
| CURSE | 저주 | 82% |
| DANCE | 춤 | 82% |
| DAWN | 새벽 | 82% |
| DEALER | 딜러 | 82% |
| DIALOGUE | 대화 | 82% |
| DIARY | 일기 | 82% |
| DIESEL | 디젤 | 82% |
| DIVERSE | 다양한 | 82% |
| DRAG | 끌다 | 82% |
| DRAW | 그리다 | 82% |
| ENDED | 끝난 | 82% |
| EYES | 눈 (신체) | 82% |
| FLAVOR | 맛 | 82% |
| GLASS | 유리 | 82% |
| GLASSES | 안경 | 82% |
| GOLD | 금 | 82% |
| HANDSOME | 잘생긴 | 82% |
| HEART | 심장 | 82% |
| HUNGER | 배고픔 | 82% |
| INTERVIEW | 인터뷰 | 82% |
| ISOLATED | 고립된 | 82% |
| JOKE | 농담 | 82% |
| LIGHT | 빛 | 82% |
| LIST | 목록 | 82% |
| LISTEN | 듣기 | 82% |
| LONG | 긴 | 82% |
| MAKEUP | 화장 | 82% |
| MASK | 마스크 | 82% |
| MOUNTAIN | 산 | 82% |
| NOISE | 소음 | 82% |
| ONLINE | 온라인 | 82% |
| OPINION | 의견 | 82% |
| PHOENIX | 불사조 | 82% |
| POLITICIAN | 정치인 | 82% |
| PORT | 항구 | 82% |
| PORTRAIT | 초상화 | 82% |
| POSITION | 위치 | 82% |
| PRIDE | 자부심 | 82% |
| QUEEN | 여왕 | 82% |
| QUIET | 조용한 | 82% |
| RANGE | 범위 | 82% |
| RING | 반지 | 82% |
| ROAD | 도로 | 82% |
| ROCKET | 로켓 | 82% |
| SECRETARY | 비서 | 82% |
| SENTENCE | 문장 | 82% |
| SOFT | 부드러운 | 82% |
| SONG | 노래 | 82% |
| SPECIES | 종 (생물 종류) | 82% |
| STAIRS | 계단 | 82% |
| STAMP | 도장 | 82% |
| SYMBOL | 상징 | 82% |
| TATTOO | 문신 | 82% |
| TECHNICAL | 기술적인 | 82% |
| TENT | 텐트 | 82% |
| THOUGHT | 생각 | 82% |
| TOURNAMENT | 토너먼트 | 82% |
| TRIBE | 부족 | 82% |
| UNIT | 단위 | 82% |
| WALL | 벽 | 82% |
| WAREHOUSE | 창고 | 82% |
| WEBSITE | 웹사이트 | 82% |
| ABSTRACT | 추상적인 | 73% |
| ACTRESS | 여배우 | 73% |
| ADORABLE | 사랑스러운 | 73% |
| APOLOGY | 사죄 | 73% |
| AUDIO | 오디오 | 73% |
| BEARD | 수염 | 73% |
| BEAUTY | 아름다움 | 73% |
| BENCH | 벤치 | 73% |
| BLADE | 칼날 | 73% |
| BLANKET | 담요 | 73% |
| BLUES | 블루스 | 73% |
| BOWL | 그릇 | 73% |
| BULL | 황소 | 73% |
| BUSINESS | 사업 | 73% |
| CEILING | 천장 | 73% |
| CIRCLE | 원 | 73% |
| CLOSED | 닫힌 | 73% |
| COLLEGE | 단과대학 | 73% |
| COLUMN | 기둥 | 73% |
| COMMITTEE | 위원회 | 73% |
| COMPANY | 회사 | 73% |
| COUCH | 소파 | 73% |
| CRITICISM | 비판 | 73% |
| DARK | 어두운 | 73% |
| DEBUT | 데뷔 | 73% |
| DEPARTURE | 출발 | 73% |
| DISASTER | 재앙 | 73% |
| DISCOUNT | 할인 | 73% |
| DOCUMENT | 문서 | 73% |
| DOOR | 문 | 73% |
| DOWNTOWN | 시내 | 73% |
| EMOTIONAL | 감정적인 | 73% |
| EXPANSION | 확장 | 73% |
| FACILITY | 시설 | 73% |
| FLIP | 뒤집다 | 73% |
| FORGET | 잊다 | 73% |
| GRAVE | 무덤 | 73% |
| HONOR | 명예 | 73% |
| JEWELRY | 보석 | 73% |
| LABORATORY | 실험실 | 73% |
| LARGE | 큰 | 73% |
| LECTURE | 강의 | 73% |
| LETTER | 편지 | 73% |
| LOGO | 로고 | 73% |
| MESSAGE | 메시지 | 73% |
| MOON | 달 | 73% |
| MOTION | 움직임 | 73% |
| MOVING | 움직이는 | 73% |
| MURDER | 살인 | 73% |
| NAVY | 해군 | 73% |
| NETWORK | 네트워크 | 73% |
| OFFICE | 사무실 | 73% |
| PAIN | 고통 | 73% |
| PAIR | 짝 | 73% |
| PARADE | 행진 | 73% |
| PATH | 길 | 73% |
| PLATFORM | 플랫폼 | 73% |
| POPE | 교황 | 73% |
| PRETTY | 예쁜 | 73% |
| PROPORTION | 함량 | 73% |
| PROVED | 증명된 | 73% |
| PUSH | 밀다 | 73% |
| RAGE | 분노 | 73% |
| RECOVER | 회복하다 | 73% |
| RECOVERY | 회복 | 73% |
| RICE | 쌀 | 73% |
| RIOT | 폭동 | 73% |
| ROOF | 지붕 | 73% |
| SCRIPT | 대본 | 73% |
| SEALED | 봉인된 | 73% |
| SEARCH | 검색 | 73% |
| SEAT | 자리 | 73% |
| SECURITY | 보안 | 73% |
| SHAPE | 모양 | 73% |
| SHORT | 짧은 | 73% |
| SING | 노래하다 | 73% |
| SINK | 가라앉다 | 73% |
| SKETCH | 스케치 | 73% |
| SMELL | 냄새 | 73% |
| SMOKE | 연기 (불타서 생기는) | 73% |
| SPACE | 공간 | 73% |
| STAY | 머물다 | 73% |
| STEM | 줄기 | 73% |
| STONE | 돌 | 73% |
| STOP | 멈추다 | 73% |
| STREET | 거리 | 73% |
| TELEVISION | 텔레비전 | 73% |
| TENSION | 긴장 | 73% |
| TESTIMONY | 증언 | 73% |
| THICK | 두꺼운 | 73% |
| TOWN | 도시 | 73% |
| TRAFFIC | 교통 | 73% |
| TRAIN | 기차 | 73% |
| TRICK | 속임수 | 73% |
| UNIVERSITY | 대학 | 73% |
| VERTICAL | 수직의 | 73% |
| VIDEO | 비디오 | 73% |
| VISIBLE | 보이는 | 73% |
| VOTE | 투표하다 | 73% |
| WAVE | 파도 | 73% |
| ACADEMY | 학원 | 64% |
| ANDROID | 안드로이드 | 64% |
| AREA | 지역 | 64% |
| ARGUMENT | 논쟁 | 64% |
| BAND | 밴드 | 64% |
| BELT | 벨트 | 64% |
| BORED | 지루한 | 64% |
| BRAND | 브랜드 | 64% |
| BUILT | 지어진 | 64% |
| BUTLER | 집사 | 64% |
| CAMPUS | 캠퍼스 | 64% |
| CARPET | 카펫 | 64% |
| CHECK | 확인하다 | 64% |
| COMMAND | 명령 | 64% |
| COMMERCIAL | 상업적인 | 64% |
| CONGRESS | 의회 | 64% |
| DIAMETER | 지름 | 64% |
| DISC | 디스크 | 64% |
| DOUBT | 의심 | 64% |
| DREW | 그렸다 | 64% |
| EMBASSY | 대사관 | 64% |
| EXIT | 출구 | 64% |
| FEEL | 느끼다 | 64% |
| FEVER | 열 | 64% |
| FIVE | 다섯 | 64% |
| FORT | 요새 | 64% |
| FRONT | 앞 | 64% |
| GALLERY | 갤러리 | 64% |
| GARAGE | 차고 | 64% |
| GUILT | 죄책감 | 64% |
| HISTORIC | 역사적인 | 64% |
| HISTORICAL | 역사의 | 64% |
| INJURED | 다친 | 64% |
| LABEL | 라벨 | 64% |
| LAYER | 층 | 64% |
| LINE | 선 (금이나 줄) | 64% |
| LONGER | 길게 | 64% |
| LOUD | 시끄러운 | 64% |
| MARCH | 3월 | 64% |
| MILES | 마일 | 64% |
| NORTH | 북쪽 | 64% |
| PALE | 창백한 | 64% |
| PANIC | 패닉 | 64% |
| PARLIAMENT | 국회 | 64% |
| PATTERN | 패턴 | 64% |
| PLAYOFFS | 플레이오프 | 64% |
| POLITICS | 정치 | 64% |
| PRAISE | 칭찬하다 | 64% |
| PRIVILEGE | 특권 | 64% |
| PROJECT | 프로젝트 | 64% |
| PULSE | 맥박 | 64% |
| PURPLE | 보라색 | 64% |
| RADAR | 레이더 | 64% |
| REPLACE | 대체하다 | 64% |
| REVISED | 수정된 | 64% |
| RISING | 떠오르는 | 64% |
| ROOM | 방 | 64% |
| ROUND | 둥근 | 64% |
| SATELLITE | 위성 | 64% |
| SCREEN | 화면 | 64% |
| SEGMENT | 부분 | 64% |
| SHIELD | 방패 | 64% |
| SHIP | 선박 | 64% |
| SHOWING | 보여주는 | 64% |
| SIGHT | 시야 | 64% |
| SIZE | 크기 | 64% |
| SLEEP | 잠 | 64% |
| SORT | 분류하다 | 64% |
| SPEECH | 연설 | 64% |
| SPIRITUAL | 영적인 | 64% |
| STAND | 서다 | 64% |
| STANDING | 서있는 | 64% |
| STATION | 역 (정거장) | 64% |
| STATUE | 동상 | 64% |
| STOMACH | 복부 | 64% |
| STORE | 가게 | 64% |
| STRENGTH | 힘 | 64% |
| STRING | 줄 | 64% |
| SUNSET | 일몰 | 64% |
| SUNSHINE | 햇빛 | 64% |
| SYSTEM | 시스템 | 64% |
| THIN | 얇은 | 64% |
| TIRED | 피곤한 | 64% |
| TOUCH | 만지다 | 64% |
| TOUR | 여행 | 64% |
| VINTAGE | 빈티지 | 64% |
| VIRTUAL | 가상의 | 64% |
| VOLUME | 부피 | 64% |
| WAGES | 임금 | 64% |
| WORRY | 걱정 | 64% |
| YARD | 야드 | 64% |
| ADVICE | 조언 | 55% |
| ARTICLE | 기사 (신문) | 55% |
| ASHAMED | 부끄러운 | 55% |
| ATTENDANCE | 출석 | 55% |
| BANNER | 배너 | 55% |
| BELL | 종 (소리내는 기구) | 55% |
| BILL | 계산서 | 55% |
| BITTER | 쓴 | 55% |
| BLANK | 빈칸 | 55% |
| BOTTOM | 아래 | 55% |
| BROADCAST | 방송하다 | 55% |
| BUSH | 덤불 | 55% |
| CABLE | 케이블 | 55% |
| CARGO | 화물 | 55% |
| CARRIER | 캐리어 | 55% |
| CEMETERY | 묘지 | 55% |
| CHART | 차트 | 55% |
| CHASE | 쫓다 | 55% |
| CLICK | 클릭하다 | 55% |
| CLIP | 클립 | 55% |
| COMPLAIN | 불평하다 | 55% |
| CONFERENCE | 회의 | 55% |
| CONFUSED | 혼란스러운 | 55% |
| COSTUME | 의상 | 55% |
| COTTON | 면직물 | 55% |
| COUPLE | 커플 | 55% |
| COURT | 법정 | 55% |
| DATA | 데이터 | 55% |
| DISEASE | 질병 | 55% |
| DROP | 떨어뜨리다 | 55% |
| ENORMOUS | 거대한 | 55% |
| ENTER | 들어가다 | 55% |
| ENTRY | 입장 | 55% |
| EPISODE | 에피소드 | 55% |
| ESSAY | 에세이 | 55% |
| EXCHANGE | 교환 | 55% |
| EXHAUSTED | 지친 | 55% |
| EXHIBITION | 전시 | 55% |
| EXPLORE | 탐험하다 | 55% |
| FALLEN | 떨어진 | 55% |
| FEEDBACK | 피드백 | 55% |
| FENCE | 울타리 | 55% |
| FICTION | 소설 | 55% |
| FIFTEEN | 열다섯 | 55% |
| FINALE | 피날레 | 55% |
| FOLLOW | 따라가다 | 55% |
| FORUM | 포럼 | 55% |
| FUNCTIONAL | 기능적인 | 55% |
| GEAR | 기어 | 55% |
| GRAPHIC | 그래픽 | 55% |
| HARD | 어려운 | 55% |
| HEIGHT | 높이 | 55% |
| ICON | 아이콘 | 55% |
| INTERIOR | 인테리어 | 55% |
| JEAN | 청바지 | 55% |
| JOURNALISM | 저널리즘 | 55% |
| LOSS | 손실 | 55% |
| MAGIC | 마법 | 55% |
| MALL | 쇼핑몰 | 55% |
| MEDIA | 미디어 | 55% |
| MILITARY | 군대 | 55% |
| MINISTER | 장관 | 55% |
| MUSICAL | 뮤지컬 | 55% |
| MYTH | 신화 | 55% |
| NOTE | 노트 | 55% |
| PAINTED | 칠해진 | 55% |
| PARALLEL | 평행한 | 55% |
| PARKING | 주차 | 55% |
| PASSENGER | 승객 | 55% |
| PENNY | 페니 | 55% |
| PERMISSION | 허락 | 55% |
| POST | 우편 | 55% |
| PROFILE | 프로필 | 55% |
| PROMISE | 약속 | 55% |
| QUANTITY | 양 (수량) | 55% |
| RACE | 경주 | 55% |
| REFLECTION | 반사 | 55% |
| ROPE | 밧줄 | 55% |
| ROYAL | 왕족의 | 55% |
| SAVING | 저축 | 55% |
| SETTING | 설정 | 55% |
| SETTLEMENT | 정착 | 55% |
| SHAME | 부끄러움 | 55% |
| SHEET | 종이 | 55% |
| SHERIFF | 보안관 | 55% |
| SHINE | 빛나다 | 55% |
| SHORTS | 반바지 | 55% |
| SHOUT | 소리지르다 | 55% |
| SILENCE | 침묵 | 55% |
| SITE | 장소 | 55% |
| SLOWLY | 느리게 | 55% |
| SMILE | 미소 | 55% |
| SPELL | 주문 | 55% |
| SPLIT | 나누다 | 55% |
| SPORTS | 스포츠 | 55% |
| STAFF | 직원 | 55% |
| SUMMIT | 정상 (꼭대기) | 55% |
| SWEAR | 맹세하다 | 55% |
| TARGET | 목표 | 55% |
| TEENAGER | 청소년 | 55% |
| TELLING | 말하는 | 55% |
| THEATER | 극장 | 55% |
| THERMAL | 열의 | 55% |
| TIED | 묶인 | 55% |
| TONE | 톤 | 55% |
| TOURIST | 관광객 | 55% |
| TOWER | 타워 | 55% |
| TREASURY | 보물 | 55% |
| UNIFORM | 유니폼 | 55% |
| VALLEY | 계곡 | 55% |
| WRITING | 글쓰기 | 55% |
| YOUTH | 젊음 | 55% |
| ACTING | 연기 (배우) | 45% |
| ADDITION | 추가 | 45% |
| AFFECTED | 영향받은 | 45% |
| AGENT | 요원 | 45% |
| AHEAD | 앞에 | 45% |
| ARENA | 경기장 | 45% |
| ARSENAL | 무기고 | 45% |
| ATHLETE | 운동선수 | 45% |
| AWKWARD | 어색한 | 45% |
| BARRIER | 장벽 | 45% |
| BASEMENT | 지하실 | 45% |
| BISHOP | 주교 | 45% |
| BLAME | 비난하다 | 45% |
| BRAINS | 뇌 | 45% |
| BRANCH | 가지 | 45% |
| CABIN | 오두막 | 45% |
| CANAL | 운하 | 45% |
| CAPACITY | 용량 | 45% |
| CELEBRITY | 유명인 | 45% |
| CHAMPION | 우승자 | 45% |
| CHEERS | 건배 | 45% |
| COLLECTION | 수집품 | 45% |
| DECK | 갑판 | 45% |
| DESCRIBE | 묘사하다 | 45% |
| DIPLOMATIC | 외교적인 | 45% |
| DIVE | 뛰어들다 | 45% |
| DOWNLOAD | 다운로드 | 45% |
| DRESS | 드레스 | 45% |
| FAIL | 실패하다 | 45% |
| FILLED | 채워진 | 45% |
| FINANCIAL | 금융의 | 45% |
| FLYING | 나는 | 45% |
| FORMATION | 형성 | 45% |
| FOUNDATION | 기초 | 45% |
| FRAMEWORK | 틀 | 45% |
| GANG | 깡패 | 45% |
| GIANT | 거인 | 45% |
| GLOBE | 지구 | 45% |
| GRID | 격자 | 45% |
| GRIEF | 슬픔 | 45% |
| HALF | 반 | 45% |
| HAPPY | 행복한 | 45% |
| HIGHLIGHT | 하이라이트 | 45% |
| HORIZON | 수평선 | 45% |
| HOST | 주인 | 45% |
| HURTING | 아픈 | 45% |
| IMAGE | 이미지 | 45% |
| LARGELY | 크게 | 45% |
| LICENSE | 면허 | 45% |
| LOWER | 낮은 | 45% |
| MAINSTREAM | 주류 | 45% |
| MARK | 표시하다 | 45% |
| MENTION | 언급 | 45% |
| MESS | 엉망 | 45% |
| MICE | 쥐 | 45% |
| MOOD | 분위기 | 45% |
| NERVE | 신경 | 45% |
| NINE | 아홉 | 45% |
| NUMEROUS | 많은 | 45% |
| OPTICAL | 광학의 | 45% |
| OUTFIT | 옷 | 45% |
| PAGE | 쪽 | 45% |
| PERCENTAGE | 퍼센트 | 45% |
| PHRASE | 구절 | 45% |
| PICTURE | 그림 | 45% |
| POINT | 점 | 45% |
| PROUD | 자랑스러운 | 45% |
| REST | 쉬다 | 45% |
| ROBIN | 울새 | 45% |
| SCAN | 스캔 | 45% |
| SENIOR | 노인 | 45% |
| SIGNATURE | 서명 | 45% |
| SKILLED | 숙련된 | 45% |
| STEEL | 철 | 45% |
| STOCKS | 주식 | 45% |
| TEMPLE | 절 | 45% |
| TERRORISM | 테러 | 45% |
| THANK | 감사 | 45% |
| THREAD | 실 | 45% |
| TICKET | 표 | 45% |
| TISSUE | 조직 | 45% |
| TRAILER | 트레일러 | 45% |
| TUBE | 관 | 45% |
| TURN | 돌다 | 45% |
| UNHAPPY | 불행한 | 45% |
| VETERAN | 베테랑 | 45% |
| VOTER | 유권자 | 45% |
| WIDESPREAD | 널리 퍼진 | 45% |
| YELLOW | 노란색 | 45% |
| YIELD | 양보하다 | 45% |
| ANNOUNCE | 발표하다 | 36% |
| ARCHIVES | 아카이브 | 36% |
| ARTWORK | 예술작품 | 36% |
| ASLEEP | 잠든 | 36% |
| AVIATION | 비행 | 36% |
| BALLOT | 투표용지 | 36% |
| BARREL | 통 | 36% |
| BEAM | 빔 | 36% |
| BELONGING | 소지품 | 36% |
| BLUE | 파랑 | 36% |
| BOARD | 보드 | 36% |
| BUILDING | 건물 | 36% |
| BUST | 터지다 | 36% |
| CAGE | 새장 | 36% |
| CALM | 차분한 | 36% |
| CAPTAIN | 대장 | 36% |
| CHAIN | 체인 | 36% |
| CHARGING | 충전 | 36% |
| CHIEF | 우두머리 | 36% |
| COLONIAL | 식민지의 | 36% |
| COMMENT | 코멘트 | 36% |
| CONCRETE | 콘크리트 | 36% |
| COPY | 복사 | 36% |
| CORNER | 구석 | 36% |
| COST | 비용 | 36% |
| COUNCIL | 위원회 | 36% |
| COVERED | 덮인 | 36% |
| CROP | 자르다 | 36% |
| CRYSTAL | 크리스탈 | 36% |
| CURVE | 커브 | 36% |
| DEALING | 거래 | 36% |
| DEAN | 학장 | 36% |
| DEBATE | 토론하다 | 36% |
| DEPRESSION | 우울증 | 36% |
| DERBY | 더비 | 36% |
| DETAILED | 자세한 | 36% |
| DISCUSSION | 논의 | 36% |
| DISPLAYED | 전시된 | 36% |
| DOUBLE | 두배의 | 36% |
| DOZEN | 다스 | 36% |
| DRINKING | 마시는 | 36% |
| ECHO | 메아리 | 36% |
| EDITING | 편집 | 36% |
| EMPLOYER | 고용주 | 36% |
| FAITH | 신념 | 36% |
| FLASH | 플래시 | 36% |
| FLEW | 날다 | 36% |
| FORMAL | 공식적인 | 36% |
| GALAXY | 우주 | 36% |
| GATE | 대문 | 36% |
| GENTLY | 부드럽게 | 36% |
| GLAD | 기쁘다 | 36% |
| GRATEFUL | 감사하는 | 36% |
| GREEN | 초록색 | 36% |
| HALL | 홀 | 36% |
| HOOK | 갈고리 | 36% |
| HULL | 선체 | 36% |
| INSPECTION | 조사 | 36% |
| INVENTORY | 재고 | 36% |
| LAMP | 램프 | 36% |
| LAYOUT | 레이아웃 | 36% |
| LEADING | 이끄는 | 36% |
| LINEAR | 선형의 | 36% |
| LINEUP | 라인업 | 36% |
| MAIL | 메일 | 36% |
| MEASURING | 측정 | 36% |
| MINES | 지뢰 | 36% |
| MONITOR | 모니터 | 36% |
| NERVOUS | 긴장한 | 36% |
| OFFERING | 제안하는 | 36% |
| PASS | 통과하다 | 36% |
| PASSAGE | 통로 | 36% |
| PATCH | 패치 | 36% |
| PAYMENT | 지불 | 36% |
| PINK | 분홍색 | 36% |
| PRIEST | 성직자 | 36% |
| PROPERTY | 소유물 | 36% |
| PUBLISHING | 출판 | 36% |
| PURSUIT | 추구 | 36% |
| QUOTE | 인용하다 | 36% |
| RAIL | 레일 | 36% |
| REMOVING | 제거하는 | 36% |
| RESIDENCE | 거주 | 36% |
| RESIDENT | 거주자 | 36% |
| RESORT | 리조트 | 36% |
| RETREAT | 후퇴하다 | 36% |
| REVIEW | 리뷰 | 36% |
| ROLLED | 말린 | 36% |
| ROTATION | 회전 | 36% |
| ROUTE | 경로 | 36% |
| RUNNER | 주자 | 36% |
| SANG | 노래부르다 | 36% |
| SCALE | 규모 | 36% |
| SCREAM | 비명 | 36% |
| SCREW | 나사 | 36% |
| SHORE | 해안가 | 36% |
| SIDE | 옆 | 36% |
| SIGN | 신호 | 36% |
| SLIM | 날씬한 | 36% |
| SLOT | 슬롯 | 36% |
| SOVIET | 소련 | 36% |
| SPOT | 지점 | 36% |
| STICK | 막대 | 36% |
| STORAGE | 저장 | 36% |
| STUNNING | 놀라운 | 36% |
| SUIT | 정장 | 36% |
| SUNNY | 화창한 | 36% |
| TALL | 키가 큰 | 36% |
| TEXT | 문자 | 36% |
| TIDE | 조류 | 36% |
| TRACE | 흔적 | 36% |
| TWIST | 꼬다 | 36% |
| VAST | 광대한 | 36% |
| VENUE | 개최지 | 36% |
| VESSEL | 혈관 | 36% |
| WAKE | 깨우다 | 36% |
| YOUNG | 젊은 | 36% |
| ACCENT | 억양 | 27% |
| ADJACENT | 인접한 | 27% |
| ADVOCATE | 옹호하다 | 27% |
| ANSWER | 답 | 27% |
| ATLANTIC | 대서양 | 27% |
| AVENUE | 대로 | 27% |
| BASE | 기반 | 27% |
| BLACK | 검정 | 27% |
| BOOT | 부츠 | 27% |
| BRASS | 황동 | 27% |
| CABINET | 캐비닛 | 27% |
| CALL | 부르다 | 27% |
| CAPE | 망토 | 27% |
| CASUAL | 일상적인 | 27% |
| CHAT | 떠들다 | 27% |
| CLASSES | 수업 | 27% |
| COACHING | 코칭 | 27% |
| COASTAL | 해안의 | 27% |
| COLLAPSE | 무너지다 | 27% |
| COLLAR | 칼라 | 27% |
| COMIC | 웃긴 | 27% |
| COMPONENT | 구성요소 | 27% |
| CONTROLLER | 조종기 | 27% |
| COPE | 다루다 | 27% |
| CROSS | 건너다 | 27% |
| CURRENCY | 화폐 | 27% |
| CUSTOM | 관습 | 27% |
| DEPUTY | 부관 | 27% |
| DISTANT | 먼 | 27% |
| EARNINGS | 수입 | 27% |
| FEAR | 공포 | 27% |
| FLEET | 함대 | 27% |
| FORM | 형성하다 | 27% |
| FORTUNE | 행운 | 27% |
| FULL | 가득찬 | 27% |
| GENDER | 성별 | 27% |
| GORGEOUS | 아름다운 | 27% |
| GOSPEL | 복음 | 27% |
| HACK | 해킹하다 | 27% |
| HEARING | 듣는 | 27% |
| HOOD | 후드 | 27% |
| JERSEY | 져지 | 27% |
| LEVEL | 단계 | 27% |
| LIABILITY | 책임 | 27% |
| LIEUTENANT | 중위 | 27% |
| LOADING | 로딩 | 27% |
| MANUAL | 매뉴얼 | 27% |
| MARINE | 해양의 | 27% |
| MECHANISM | 작동원리 | 27% |
| MEETING | 만남 | 27% |
| MILLIONS | 수백만 | 27% |
| NARRATIVE | 서사 | 27% |
| NOTICE | 알림 | 27% |
| PACK | 무리 | 27% |
| PATROL | 순찰하다 | 27% |
| PEAK | 꼭대기 | 27% |
| PERSONNEL | 인사과 | 27% |
| PETITION | 청원 | 27% |
| PILE | 쌓다 | 27% |
| POSE | 포즈 | 27% |
| PRESSING | 압박하는 | 27% |
| PRINCIPAL | 교장 | 27% |
| PRINT | 인쇄하다 | 27% |
| PROGRESS | 진행 | 27% |
| PROPAGANDA | 프로파간다 | 27% |
| RAISED | 길러진 | 27% |
| RALLY | 랠리 | 27% |
| REBEL | 반란 | 27% |
| RECORDING | 녹화 | 27% |
| RELIEF | 안도 | 27% |
| REPLY | 답장 | 27% |
| REPORT | 보고서 | 27% |
| RIDGE | 산등성이 | 27% |
| ROSTER | 명단 | 27% |
| SALE | 세일 | 27% |
| SEQUENCE | 순서 | 27% |
| SHADE | 그늘 | 27% |
| SPEAKER | 화자 | 27% |
| SQUARE | 사각형 | 27% |
| STACK | 더미 | 27% |
| STICKS | 막대기 | 27% |
| STRAIGHT | 똑바른 | 27% |
| STRANGER | 이방인 | 27% |
| STREAMING | 스트리밍 | 27% |
| SUITE | 스위트룸 | 27% |
| SWORD | 검 | 27% |
| TABLE | 식탁 | 27% |
| THEFT | 도둑 | 27% |
| THESIS | 논문 | 27% |
| TRACK | 추적하다 | 27% |
| VIEW | 경치 | 27% |
| VISION | 시력 | 27% |
| WALKER | 보행자 | 27% |
| WHITE | 하얀 | 27% |
| WIRE | 와이어 | 27% |
| WITNESS | 목격자 | 27% |
| ADMITTED | 인정된 | 18% |
| ADVANCE | 전진 | 18% |
| AFRAID | 두려운 | 18% |
| AGING | 노화 | 18% |
| ANXIETY | 불안 | 18% |
| ASSEMBLY | 모임 | 18% |
| BANG | 펑 | 18% |
| BOOM | 붐 | 18% |
| BREED | 기르다 | 18% |
| BULK | 대량 | 18% |
| BUZZ | 윙윙거리다 | 18% |
| CIVILIAN | 민간인 | 18% |
| CLEAR | 깨끗한 | 18% |
| COMMENTARY | 논평 | 18% |
| DISORDER | 무질서 | 18% |
| DISPUTE | 분쟁 | 18% |
| EDITORIAL | 사설 | 18% |
| ELDER | 나이가 많은 | 18% |
| FABRIC | 섬유 | 18% |
| FIGURE | 수치 | 18% |
| FILM | 필름 | 18% |
| FLED | 도망친 | 18% |
| FOOTAGE | 자료화면 | 18% |
| GOVERNOR | 통치자 | 18% |
| HANDFUL | 한 줌의 | 18% |
| HANGING | 매달린 | 18% |
| HEALING | 치료 | 18% |
| HOUSING | 주택 | 18% |
| HUGE | 막대한 | 18% |
| INQUIRY | 문의 | 18% |
| JOURNAL | 저널 | 18% |
| LIGHTING | 조명 | 18% |
| LITTLE | 적은 | 18% |
| MEMORIAL | 기념의 | 18% |
| MOMENTUM | 운동량 | 18% |
| OFFICER | 회사원 | 18% |
| ORDERED | 주문된 | 18% |
| OVERVIEW | 개요 | 18% |
| PACKAGE | 패키지 | 18% |
| POLE | 장대 | 18% |
| RECEPTION | 접수처 | 18% |
| SHED | 비추다 | 18% |
| SILENT | 고요한 | 18% |
| SQUAD | 분대 | 18% |
| STOOD | 서있다 | 18% |
| TASTE | 맛보다 | 18% |
| URGE | 욕구 | 18% |
| VISUAL | 시각적인 | 18% |
| VOCAL | 목소리의 | 18% |
| WATCH | 지켜보다 | 18% |
| WEARING | 옷을 입은 | 18% |
| WITHDRAWAL | 철회 | 18% |
| ARMY | 육군 | 9% |
| CONCERN | 염려하다 | 9% |
| DIVISION | 분배 | 9% |
| FIRM | 견고한 | 9% |
| FURTHER | 나아가 | 9% |
| GRIP | 움켜쥐다 | 9% |
| HOLD | 들다 | 9% |
| LANE | 차선 | 9% |
| ROCKY | 바위의 | 9% |
| TALK | 이야기하다 | 9% |
| TRAIL | 자취 | 9% |
| TUNE | 선율 | 9% |
| WIDE | 넓다 | 9% |
| CHAMBER | 회의실 | 0% |
| LODGE | 산장 | 0% |
| PISSED | 열받은 | 0% |

# Instruction presented to participants.

(1) The instruction for English-speaking participants.

| Thank you for participating in the survey.  In the survey, you will see a word and evaluate the word in various terms.  Please rate your thoughts on each item. There is no right or wrong, so please use your own judgement. Also, it is better to rate intuitively based on the first thought that comes to mind rather than thinking too carefully.  For example, you would rate a word 'COMPUTER' as below:  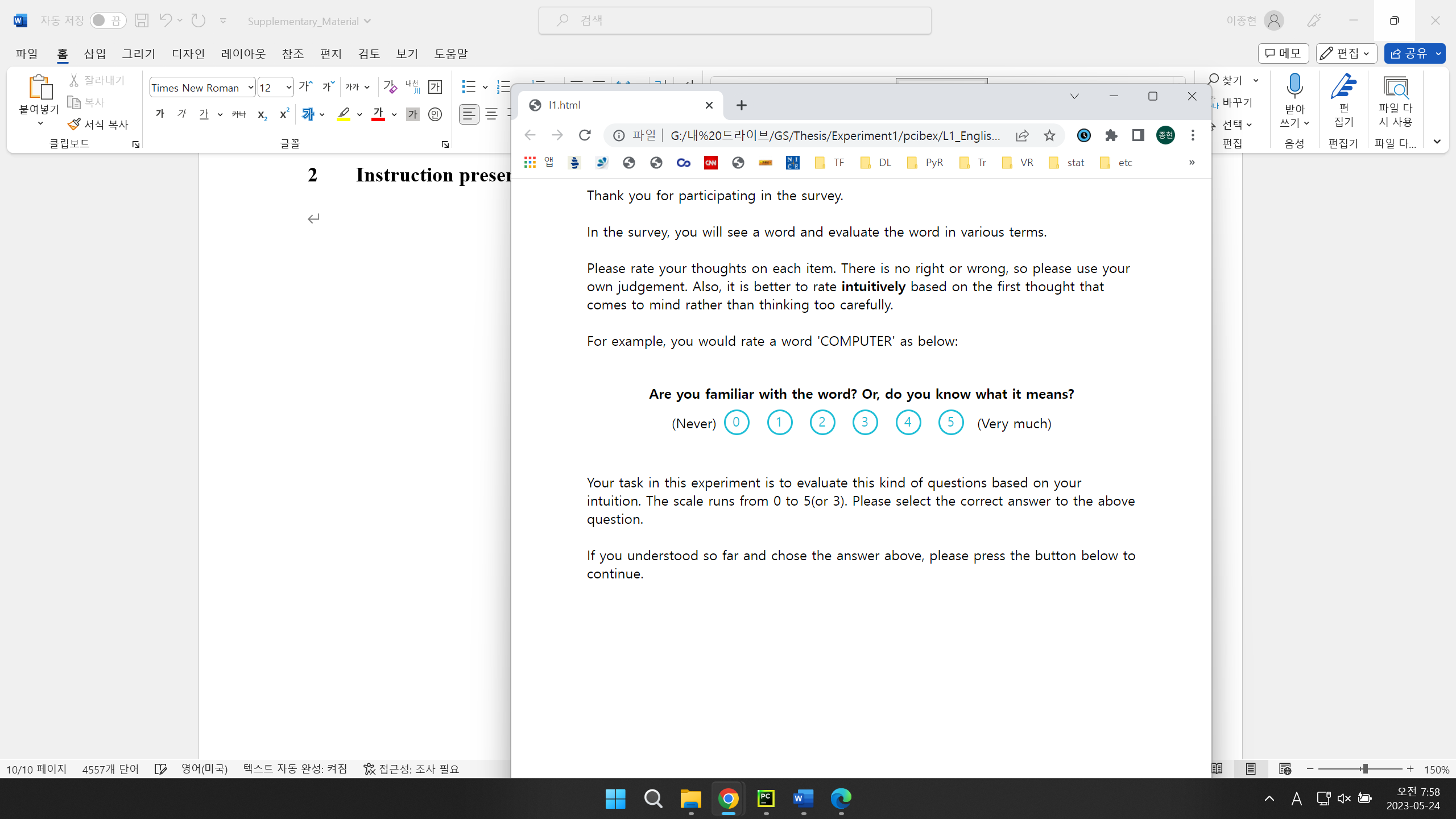  Your task in this experiment is to evaluate this kind of questions based on your intuition. The scale runs from 0 to 5(or 3). Please select the correct answer to the above question.  If you understood so far and chose the answer above, please press the button below to continue. |
| --- |
| From now on, we will explain in detail the items to be evaluated.  First, you will be asked to rate how much you experience everyday concepts using six different perceptual senses.  As an example, consider evaluating the concept, “COMPUTER”. You will experience this concept using a variety of senses. It could be the vision of looking at the screen, or the auditory sense of the sound. It could also be an internal sensation in your body that you feel while seeing and hearing it. You may experience this concept using one or two senses, or you may use multiple senses. Or it may not use any senses.  The six senses to be evaluated are sight, hearing, smell, taste, touch, and the sense of internal organs. Please rate each sensation independently. You don't have to choose one of the six senses. Multiple senses may be used for a word, or none of the senses may be used at all. The scale ranges from 0 (not experienced at all with that sense) to 5 (experienced greatly with that sense). Click on a number to select a rating for each scale, then click the "Continue" button to move to the next evaluation.  (*Of these, internal organ sensations refer to the one felt through receptors inside the body. Examples are body temperature, hunger, thirst, digestion, and heartbeat.) |
| Next, you will assess on your familiarity with certain words.  How familiar are you with the above words? Or, how much do you know what it means? (Unfamiliar 0 - Very Familiar 3)  The scale is from 0 to 3. Select a number for the scale by clicking on the number and then click the "Next" button to move to the next word to be evaluated.  If you understand so far, check the box below and click the "Start" button to start the experiment. The experiment starts with practice items. |

(2) The instruction for Korean-speaking participants.

| 안녕하세요. 실험에 참여해주셔서 감사합니다.  지금부터 여러분은 특정 개념을 나타내는 영어 단어를 보고 그 단어에 대해서 다양한 항목으로 평가하게 되실 것입니다.  각 항목에 대한 여러분의 생각을 평가해주세요. 옳고 그름은 없으니 각자의 기준에 따라 판단하시기 바랍니다. 또한 지나치게 신중하게 고민하는 것보다는 가장 먼저 떠오른 생각을 기준으로 직관적으로 판단하는 것이 더 좋습니다.  예를 들어, 여러분은 영어 단어 '컴퓨터'에 대해서 다음과 같은 항목을 평가하게 됩니다.  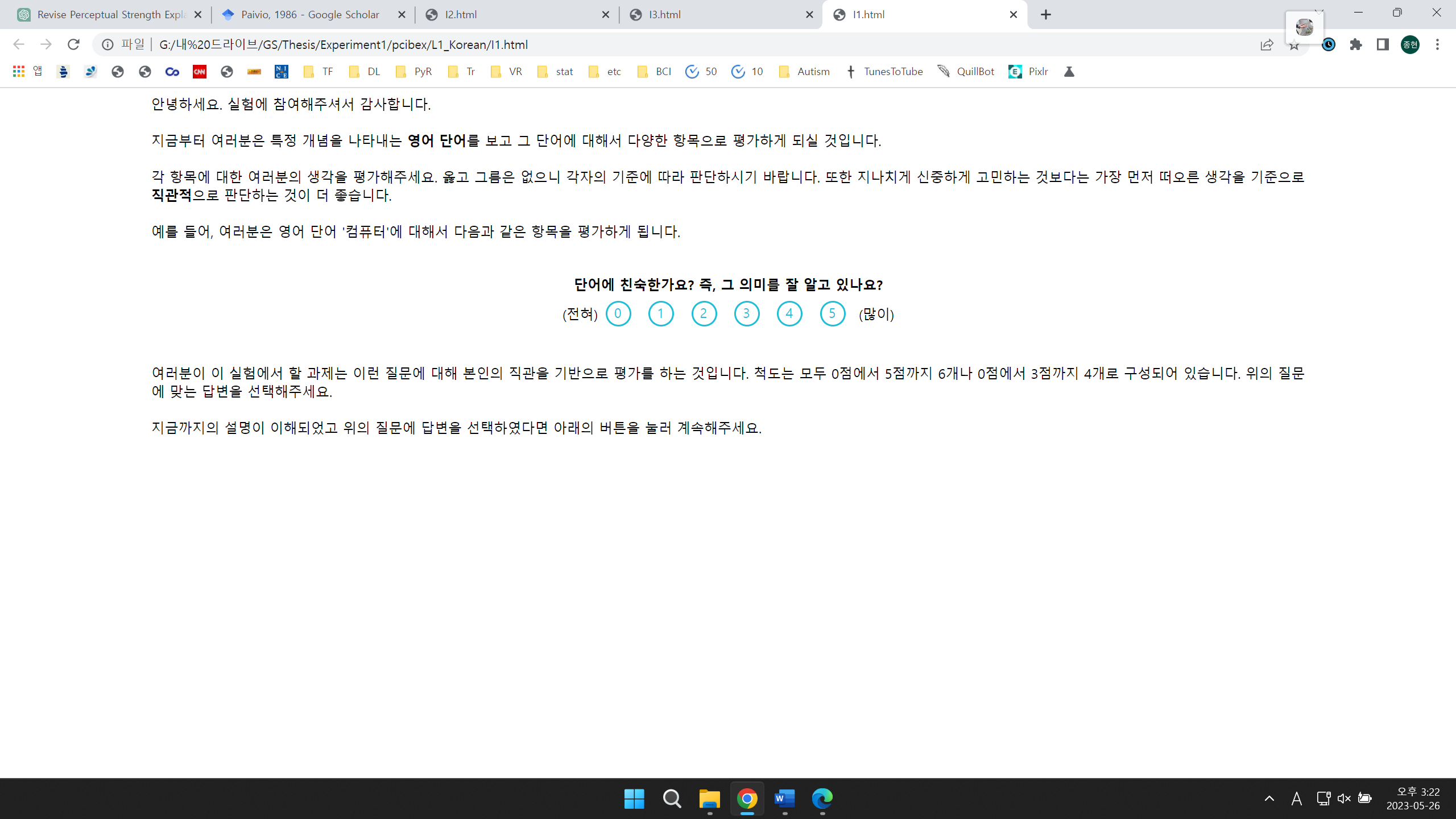  여러분이 이 실험에서 할 과제는 이런 질문에 대해 본인의 직관을 기반으로 평가를 하는 것입니다. 척도는 모두 0점에서 5점까지 6개나 0점에서 3점까지 4개로 구성되어 있습니다. 위의 질문에 맞는 답변을 선택해주세요.  지금까지의 설명이 이해되었고 위의 질문에 답변을 선택하였다면 아래의 버튼을 눌러 계속해주세요. |
| --- |
| 지금 부터 평가할 항목에 대해서 구체적으로 설명드리겠습니다.  먼저, 여러분은 특정 개념(단어)을 경험하는 데 있어서 6가지 다른 감각을 얼마나 많이 사용하는지를 평가하게 될 것입니다.  다시 예를 들어, “컴퓨터”라는 개념을 평가한다고 생각해보세요. 여러분은 다양한 감각을 사용하여 이 개념을 경험할 것입니다. 그것은 화면을 바라보는 시각일 수도 있고, 소리를 듣는 청각일 수도 있습니다. 그것을 보고 듣는 동안 느껴지는 몸의 내부 감각일 수도 있습니다. 여러분은 한두개의 감각을 이용하여 이 개념을 경험할 수도 있고 여러 개의 감각을 이용할 수도 있습니다. 혹은 어떤 감각도 이용하지 않을 수도 있습니다.  평가할 6가지의 감각은, 시각, 청각, 후각, 미각, 촉각, 내부 기관 감각입니다. 각각의 감각에 대해서 독립적으로 평가해주세요. 6가지의 감각 중 하나를 선택해야하는 것이 아닙니다. 한 단어에 대해 여러개의 감각의 이용될 수도 있고 전혀 이용되지 않을 수도 있습니다. 척도는 0(그 감각에 대해 전혀 경험하지 않음)에서 5(그 감각에 대해 많이 경험함)까지입니다. 숫자를 클릭하여 각 척도에 대한 점수를 선택한 후 "Continue" 버튼을 클릭하면 다음 평가항목으로 이동합니다.  (*이 중 내부 기관 감각은 몸 내부의 수용기관을 통해서 느껴지는 감각을 의미합니다. 체온, 배고픔, 목마름, 소화, 심장박동 등이 이런 감각의 예시입니다.) |
| 또한, 여러분은 특정 개념(단어)에 대한 친숙도에 대해서 평가하게 될 것입니다.  위의 단어에 얼마나 친숙한가요? 즉, 그 의미를 얼마나 알고 있나요? (친숙하지 않음 0 - 매우 친숙함 3)  척도는 0에서 3까지입니다. 숫자를 클릭하여 각 척도에 대한 점수를 선택한 후 "Next" 버튼을 클릭하면 평가할 다음 단어로 이동합니다.  지금까지의 설명이 이해되었다면 아래에 체크하시고 "Start" 버튼을 눌러 실험을 시작해주세요. 실험은 연습문항부터 시작됩니다. |

(3) Examples of Assessment Display Presented to Participants


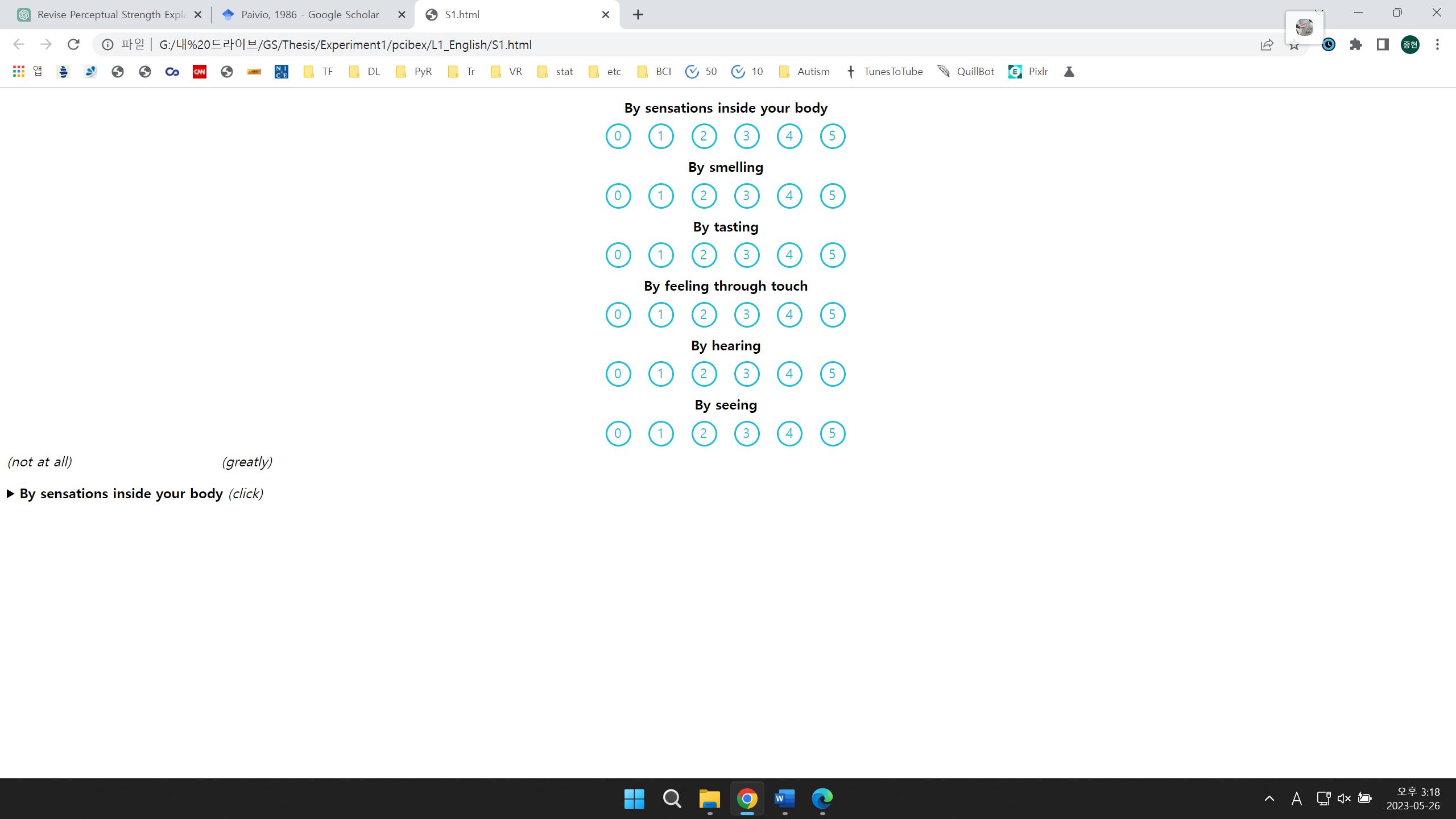

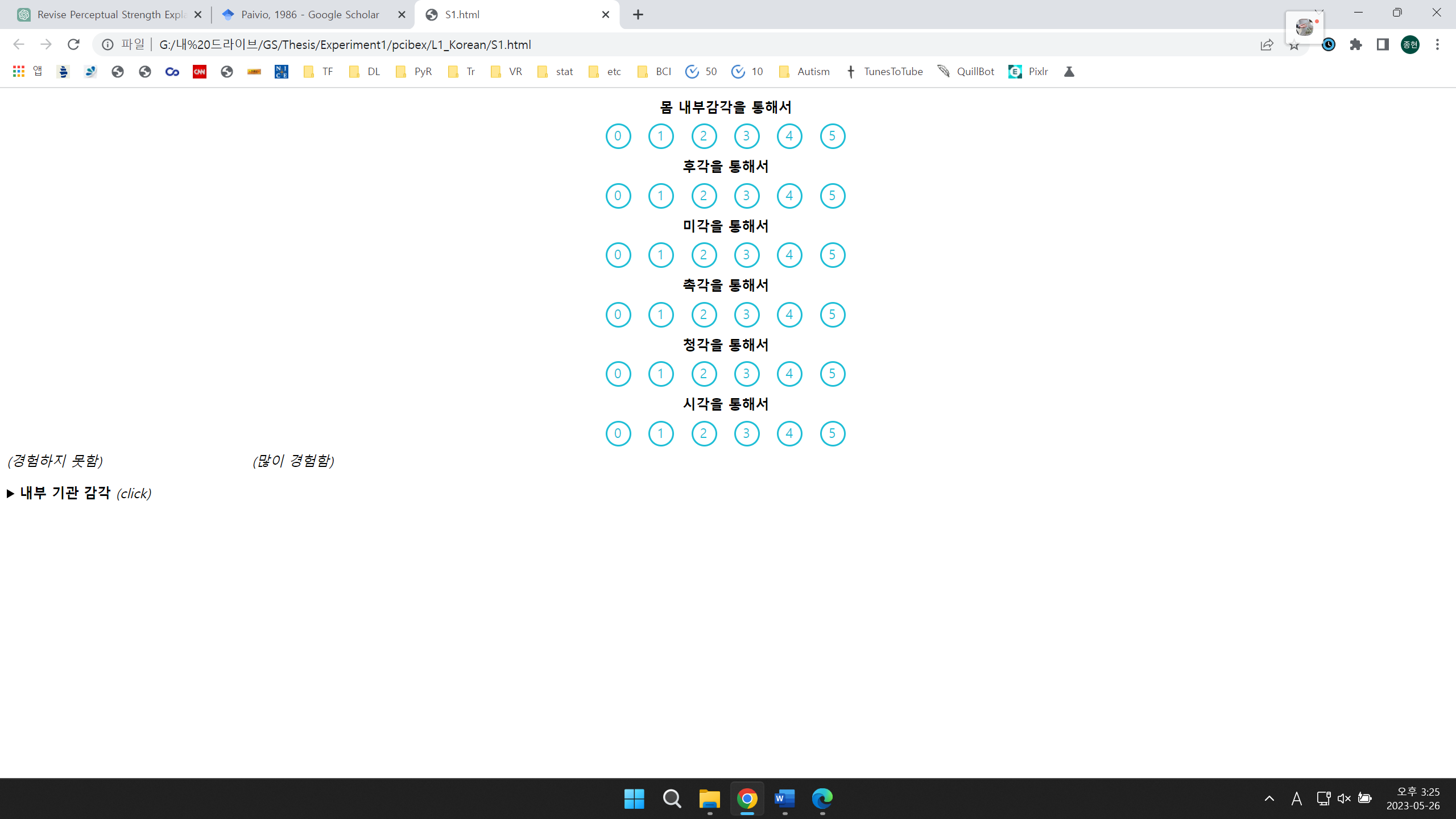

Supplement: Supplementary file 1 [file Data_Sheet_1.docx]
